# Vagus Nerve Stimulation differentially modulates P3b in responders and non-responders: toward a biomarker of therapeutic efficacy

**DOI:** 10.3389/fnins.2026.1786262

**Published:** 2026-06-17

**Authors:** Marie Dawant, Ana Marta Dias, Venethia Danthine, Jean Delbeke, Marianne de Tourtchaninoff, Riëm El Tahry, Antoine Nonclercq

**Affiliations:** 1 Bio- Electro- and Mechanical Systems (BEAMS), Ecole Polytechnique de Bruxelles, Université libre de Bruxelles, Brussels, Belgium; 2 Department of Clinical Neuroscience, Institute of Neuroscience (IoNS), Université Catholique de Louvain, Brussels, Belgium; 3 Center for Refractory Epilepsy, Department of Neurology, Cliniques Universitaires Saint-Luc (CUSL), Brussels, Belgium

**Keywords:** biomarkers, drug-resistant epilepsy, locus coeruleus-noradrenergic system, neuromodulation, P3b event-related potential, vagus nerve stimulation

## Abstract

**Introduction:**

Although vagus nerve stimulation (VNS) is an established therapy for drug-resistant epilepsy, its mechanisms of action remain unresolved, resulting in variable clinical efficacy. Given the strong anatomical and functional coupling between vagal afferents and the locus coeruleus–noradrenergic system, this study investigated whether VNS directly impacts an electrophysiological marker of this system, the P3b event-related potential, and how such modulation relates to therapeutic outcomes.

**Methods:**

Fifteen adults who had undergone long-term VNS implantation performed an auditory oddball task with the device disabled (OFF) and enabled (ON), with ON separated into electrical pulse-train (ON HIGH) and inter-burst break (ON LOW) phases to investigate the direct impact of electrical stimulation on the P3b.

**Results:**

Linear mixed-effects modelling revealed a significant interaction between VNS condition and clinical response: responders showed reduced P3b amplitude (*p* = 0.023) and prolonged latency during ON (*p* = 0.007), whereas non-responders exhibited increased amplitude (*p* = 0.009) and trending shortened latency (*p* = 0.078). These VNS-induced changes correlated monotonically with a continuous clinical response score (r_amplitude = −0.62, r_latency = 0.48). In addition, a simple classification approach based on a composite amplitude-to-latency index was included to illustrate the potential of P3b modulation as a biomarker for distinguishing responders from non-responders, showing an overall accuracy of 86.7%. No pulse-locked modulation was observed between ON HIGH and ON LOW.

**Discussion:**

These findings demonstrate that VNS elicits group-specific acute effects on cognitive–electrophysiological markers and support P3b modulation as a promising biomarker for predicting therapeutic efficacy.

## 1 Introduction

The global prevalence of epilepsy is estimated to range between 4 and 10 cases per 1,000 people (Brodie et al., 2012), with an incidence of 49 to 139 per 100,000, depending on the income of the country (World Health Organization, 2024). This represents a global population of more than 50 million patients, making epilepsy one of the most common neurological diseases (World Health Organization, 2024). If anti-seizure medication (ASMs) can control seizures in 70% of clinically treated patients, the remaining 30% are considered to suffer from drug-resistant epilepsy (DRE) (Wilcox et al., 2013). When medication is proven to be ineffective, adjunctive therapies such as surgery and neuromodulation devices can be considered (Ben-Menachem, 2002).

Since its approval, Vagus Nerve Stimulation (VNS) has proven to be a well-tolerated, non-pharmacological adjunctive treatment that, to date, has been applied to over 125,000 patients (Fetzer et al., 2021). The corresponding neuromodulation device includes a spiral electrode, implanted at the cervical level, for delivering intermittent pulse-train stimulation. One-third of patients experience a reduction in seizure frequency of more than 50%, while the other two-thirds show a partial reduction (30 to 50%) or a slight to no reduction (<30%).

Little is known about the antiepileptic effect of VNS in epilepsy (Carron et al., 2023). In this regard, identifying biomarkers that can predict the response to VNS before implantation could pave the way for new therapeutic strategies. This finding would enable more precise patient selection, reducing the incidence of non-responders. Additionally, it could improve VNS parameter adjustments, which currently rely on trial-and-error, often leading to excessive current delivery, adverse effects, and prolonged titration periods, leaving patients at risk for unpredictable seizures and associated complications. From a more fundamental perspective, it would also provide insights into the mechanisms of action of this therapy. While such biomarkers would ideally support pre-implantation selection, post-implantation classification remains clinically valuable, as it could offer an objective titration toward more effective stimulation in responders and help identify early non-responders who may benefit from alternative therapeutic strategies.

One of the hypothesized mechanisms of action is a neuromodulatory effect of VNS on the projection of the vagus nerve to the nucleus of the tractus solitarius (Thompson et al., 2019), leading to an activation of the Locus Coeruleus (LC), which is the major source of Norepinephrine (NE) in the brain (Groves et al., 2005; Sara, 2009). Animal studies have shown that VNS directly modulates LC neuronal activity. In healthy anesthetized rats, acute VNS not only elicits a direct response from isolated LC-NE neurons (Groves et al., 2005) and increases firing rates in proportion to stimulation frequency and intensity (Hulsey et al., 2017), but also enhances spontaneous firing and the proportion of neurons exhibiting burst activity (Manta et al., 2009a). An increase in extracellular NE concentrations has also been observed in the cortex and hippocampus immediately following VNS (Roosevelt et al., 2006). Additionally, the long-term effects of VNS have been highlighted by a progressive increase in the basal firing rates of NE neurons in the LC along stimulation days (Dorr and Debonnel, 2006).

In parallel with the modulatory effect of VNS on LC neuronal activity, converging evidence indicates that NE transmission plays a key role in antiepileptogenesis. Pharmacologically reduced NE levels lower the seizure threshold (Szot et al., 1999), and damage to NE pathways increases seizure susceptibility (Mishira et al., 1994). Finally, in line with its broad anatomical connections, the LC seems to play a critical role in VNS-induced seizure suppression, as lesioning the LC diminishes the anticonvulsant effects of VNS in epileptic rats (Krahl et al., 1998).

Alongside its role in the antiepileptic effect of VNS, LC neuronal activity is also a reliable indicator of the arousal state. Alterations in high-frequency phasic LC discharge rate precede changes in behavioral state in different animal models (Hobson et al., 1975; Foote et al., 1980; Aston-Jones and Bloom, 1981). In humans, in tests of attention, LC plays a role in regulating task engagement and optimizing performance (Minzenberg et al., 2008; Greene et al., 2009). Typical tasks and tests include event-related potentials (ERP), such as P300, which is subdivided into P3a and P3b components. The P3b is a positive deflection in the electroencephalogram (EEG) signal, occurring approximately 300 to 600 ms after the presentation of infrequent and deviant target stimuli during an experimental protocol called the “oddball paradigm.” In humans, P3b is thought to represent a cortical electrophysiological correlate of the phasic LC response. Indeed, in primates, the simultaneous recording of LC neurons and ERP (analogous to those in humans activity) revealed that they were elicited by the same stimuli and followed a similar time course (Aston-Jones et al., 1991). These results were supported by lesion (Pineda et al., 1989) and pharmacological studies (Swick et al., 1994), suggesting a causal contribution of LC-NE to the P300.

These findings converge as VNS has been shown to induce a dose-dependent increase in arousal and shift in behavioral state (Collins et al., 2021). Using a marker of arousal in humans, such as the P300, holds promise as a valuable tool for assessing VNS modulation of human brain activity. Previous investigations have shown that future responders have a lower P300 amplitude compared to non-responders before therapy (Hödl et al., 2020). Interestingly, following therapy, this difference disappears, and a decrease in amplitude was observed in responders only after the device was disabled (De Taeye et al., 2014). However, to date, the literature investigating P300 as a biomarker has focused on long-term recordings with and without VNS, while the brain response directly following stimulation pulses has not been explored. Although not investigated in epilepsy, brief bursts of VNS coupled with rehabilitation training significantly improve motor function recovery in patients after stroke (Engineer et al., 2019; Dawson et al., 2021; Francisco et al., 2023), suggesting that VNS may directly engage neuromodulatory networks.

This paper aims to provide additional insights into the neuromodulatory impact of VNS on the LC-NE, supporting its use as a biomarker of response to therapy. More specifically, we aim to determine whether VNS alters P3b amplitude and latency, and if these changes reflect the patient’s long-term clinical outcome. Besides, by dissecting pulse-train and inter-burst periods, we explore whether VNS acts through rapid, pulse-locked effects or through broader state-dependent neuromodulatory shifts. Finally, to identify whether these changes constitute a biomarker of long-term response, we assess their clinical utility by providing a proof-of-concept classification of responders and non-responders.

## 2 Materials and methods

### 2.1 Participants

The study was approved by the Comité d’Ethique Hospitalo-Facultaire des Cliniques Universitaires Saint-Luc (Reference No. 2022/10JAN/009) in accordance with the Declaration of Helsinki. Participants were recruited from the Center for Refractory Epilepsy at Saint-Luc University Hospital, Brussels, Belgium. They met the following criteria: 1. VNS implantation at a minimum of 18 months before the experiment, 2. Aged 18 years or older, 3. No modification of ASMs 6 months before the experiment, 4. No major secondary effect or depression due to the epileptic condition, according to clinicians’ criteria.

After approval and signature of informed consent, 17 individuals (8 female, 9 male) diagnosed with DRE and treated with VNS participated in the study. All participants were equipped with a standard VNS system comprising an invasively implanted pulse generator connected to a cervical cuff around the vagus nerve. Two patients were excluded due to poor performance in the oddball task and a technical issue. The final experimental sample pool consisted of 7 responders and 8 non-responders, following a binary classification scale (respectively, >50% or ≤50% seizure reduction). Seizure reduction was defined as the ratio of seizure frequency during the last three months before the experimental session to that during the reference period three months before VNS implantation. Moreover, the Clinical-Research Response Scale (CRRS), a classification method developed within the department and shown to be more sensitive for characterizing VNS response, was used (Danthine et al., 2025). The scale evaluates multiple clinical dimensions, including impact on seizure frequency, intensity, and duration, as well as postictal recovery and magnet responsiveness. The complete CRRS scoring, along with the test results, is reported in Supplementary material 1. Demographic and clinical characteristics of the cohort are extensively described in Table 1.

**Table 1:** Clinical and demographic characteristics of the experimental cohort.

ID	Age	Sex^a^	Epilepsy duration [years]	Epilepsy type^b^	AMSs^c^	VNS clinical parameters					VNS duration [years]	VNS model	Seizure reduction [%]	Binary response	CRRS
1	57	M	40	F	CBZ, LCM	1	20	250	30	300	18	DemiPulse Model 103	100	R	15
2	33	F	22	G	LEV, LTG	1.5	30	250	30	300	9	DemiPulse Model 103	100	R	15
3	31	F	14	G	LEV, LTG	1.5	20	250	30	300	3	AspireSR 106	80	R	15
4	36	F	29	PF	LCM, PRL	2	30	250	14	66	3	AspireSR 106	75	R	13
5	24	F	18	F	VPA	1.5	30	500	30	300	4	AspireSR 106	60	R	12
6	39	M	36	G	LTG, VPA	1.5	30	250	30	300	10	DemiPulse Model 103	100	R	13
7	22	F	6	G	LEV, LTG, TPM	1.625	30	500	30	300	1.5	SenTiva M1000	100	R	15
8	49	F	23	F	LEV, LTG, PGB	1.25	20	250	30	300	2	AspireSR 106	50	NR	12
9	47	M	41	F	BRM, CBZ, LTG	1.5	20	250	30	300	8	AspireSR 106	0	NR	6
10	51	F	21	F	CNB, LEV, LCM, PGB	1.875	20	250	30	300	3	AspireSR 106	10	NR	7
11	36	M	11	F, P	BRM, CBZ, CLB	2	30	250	21	108	2	AspireSR 106	30	NR	9
12	40	M	30	FP	CBZ, CLB, LTG	2	30	250	30	108	15	AspireSR 106	40	NR	7
13	29	M	13	G/FF	LEV, LCM, TPM	1.75	30	250	21	108	2	SenTiva M1000	0	NR	2
14	24	M	17	F	BRM, CLB, LCM	1.5	20	250	30	300	2	SenTiva M1000	50	NR	11
15	66	F	57	F, P	LEV, LCM	1.25	30	500	30	300	1.5	SenTiva M1000	40	NR	12

^a^Sex is defined as sex attributed at birth.

^b^Epilepsy types: F, focal; G, generalized.

^c^ASMs: BRM, brivaracétam; CBZ, carbamazépine; CLB, clobazam; CNB, cénobamate; LCM, lacosamide; LEV, levitaceram; LTG, lamotrigine; PGB, pregabaline; PRL, perampel; TPM, topiramate; VPA, valporate.

### 2.2 Oddball task and experimental procedure

P300 was elicited through a typical auditory oddball paradigm, consisting of a sequence of low-pitched sound stimuli embedded with high-pitched sounds, labeled as targets and non-targets, respectively. The active recognition, involving a motor response to the target, elicits the P3b, the phasic component of the P300.

**Figure 1:**
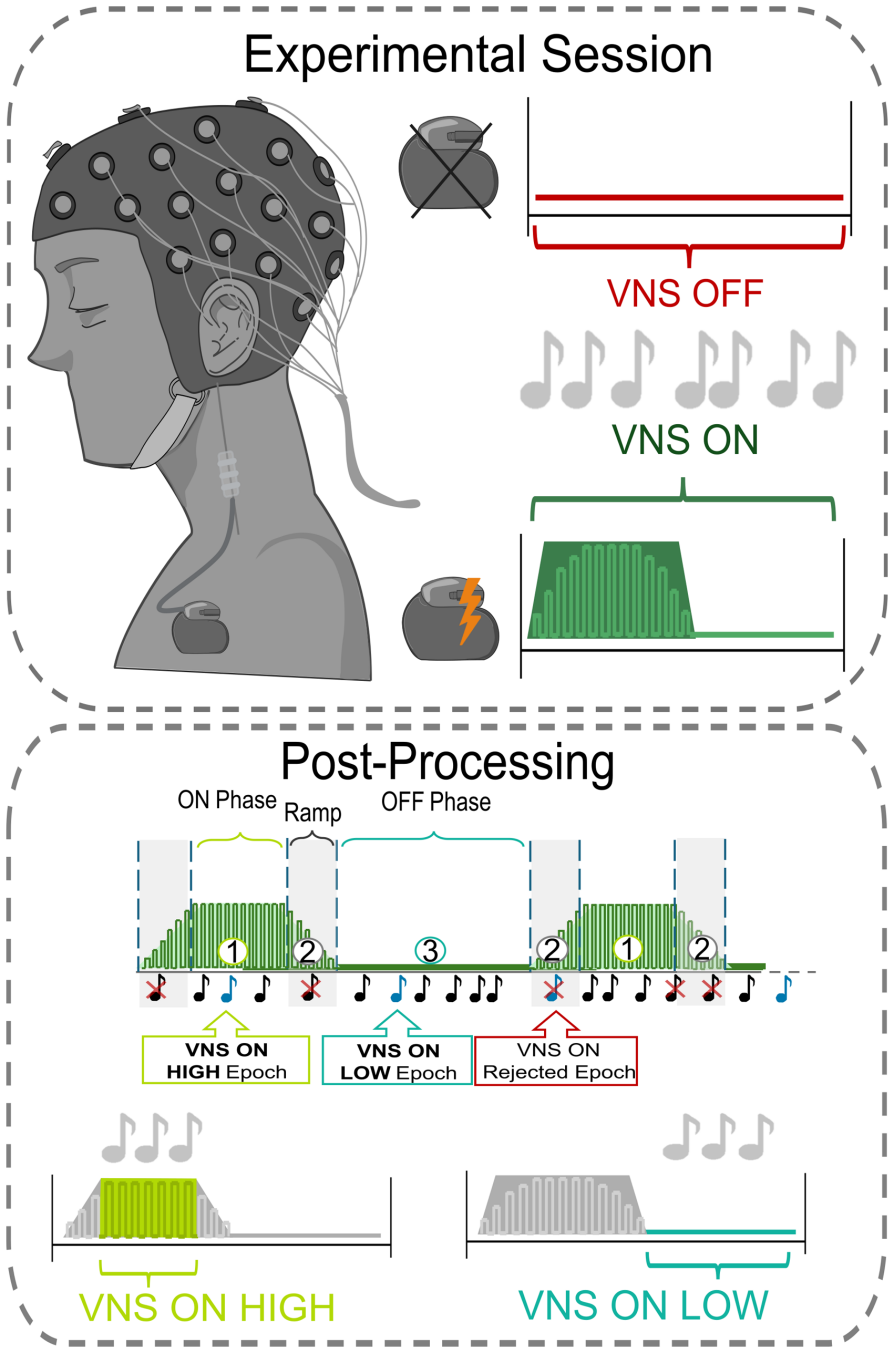
Schematic of experimental tasks and conditions. During the experimental session, patients performed an auditory oddball task under two conditions: VNS OFF and VNS ON. In post-processing, the VNS ON condition was segmented into VNS ON HIGH and VNS ON LOW based on VNS stimulation state. Epochs recorded during the effective electrical stimulation (1) were classified as VNS ON HIGH, and epochs occurring during intermittent breaks (3) as VNS ON LOW. Epochs falling within the ramp phase were excluded from the VNS ON HIGH/LOW categorization but retained in the VNS ON dataset.

Experimental conditions are schematized in Figure 1. During the experimental session, participants performed an auditory oddball task under two randomized and counterbalanced conditions: VNS OFF and VNS ON, corresponding to the device disabled and enabled, respectively, as illustrated in red and dark green in Figure 1. Auditory stimuli (target and non-target tones) were continuously presented across all conditions. In the VNS ON condition, electrical stimulation was delivered in intermittent pulse trains. This condition can be further subdivided into three distinct phases: a high intensity plateau during which the effective electrical stimulation is delivered at the programmed clinical intensity (further referred to as VNS ON HIGH), a ramp up/ramp down phase, during which pulse amplitude progressively increases or decreases, and an OFF phase, during which no stimulation is delivered (further referred to as VNS ON LOW). These phases are illustrated in Figure 1 and are labeled 1, 2, and 3, respectively. This results in alternating periods of active stimulation (VNS ON HIGH, in light green in Figure 1) and inter-burst intervals (VNS ON LOW, in turquoise in Figure 1).

During VNS ON, stimulation parameters intensity, frequency, and pulse width were identical to the patient’s clinical settings, as reported in Table 1. For all participants, the duty cycle was modified to 14 s ON and 18 s OFF, increasing the effective stimulation percentage to 56% by duty cycle. This modification was introduced to limit the experimental session to 1 h to maintain the subject’s attention. Each participant received a 1-min training session, and VNS OFF condition testing began 20 min after the VNS device was deactivated.

The auditory stimuli were pure sine waves of 300 ms duration, separated by an interstimulus interval of 1,200 ms from end to onset. The target sounds, at 2500 Hz, were distributed pseudo-randomly with a 10% probability among the standard tones at 800 Hz. The stimuli were delivered through speakers (Pulse 285, Labtec, Vancouver, United States of America), and the participants were asked to respond to the target by pressing a button. The button interface, along with all the other components necessary for this experiment, was developed in-house. During the VNS OFF condition, the oddball task consisted of two trials, each 5 min long, for a total of 400 stimuli (40 targets and 360 non-targets). During the VNS ON condition, the task consisted of 5 trials of 4 min and 30 s each, for a total of 80 targets and 720 non-targets. To investigate the acute effects of VNS (i.e., during stimulation pulses), the oddball task for the ON condition was designed to include the same number of target stimuli during VNS ON HIGH and VNS ON LOW. Auditory stimuli delivered during the effective electrical stimulation phase and intermittent breaks were, respectively, labeled as VNS ON HIGH and VNS ON LOW during post processing as illustrated in Figure 1, creating two virtual experimental conditions. All the oddball sessions were managed by a custom-written MATLAB script (version R2023b).

Participants were asked to refrain from consuming coffee, tea, energy drinks, and nicotine for at least 8 h before the experiment and to minimize head and eye movements during the recordings. Additionally, each trial began upon patient approval, after being instructed to focus on accurate target detection rather than on response speed.

### 2.3 Data collection

The recording procedure followed the guidelines and recommended methods for obtaining ERP in clinical research (Picton et al., 2000; Groves et al., 2005; Luck, 2014). The acquisition chain comprises a Biosemi recording setup (ActiveTwo, Biosemi B. V., Amsterdam, Netherlands) using 64 Ag-AgCl electrodes placed according to the standard 10–10 positions. EEG recordings were obtained non-invasively at the scalp and were purely passive, ensuring they did not influence or interfere with the functioning of the implanted VNS system. In addition, eight channels collected the VNS signal, the single-lead electrocardiogram (ECG), the horizontal electrooculogram (HEOG), and a mastoid derivation. Active electrodes were used together with a driven-right-leg circuit to minimize interferences (Nonclercq and Mathys, 2004), and the direct current (DC) offset of the electrodes was maintained below ±20 mV during the recording sessions. The data were collected at a sampling frequency of 8,192 Hz, then filtered from DC to 1,600 Hz and digitized with 24-bit resolution. A detailed presentation and assessment of the developed setup can be found in (Dawant et al., 2025).

### 2.4 Data post-processing

The raw EEG data were exported from the BDF file format into MATLAB using EEGLAB (version 2024) using the pop_biosig() function. A series of post-processing steps was applied offline using EEGLAB under MATLAB, starting with re-referencing to the average of 55 electrodes. The frontal electrodes (AFz, AF3, AF4, AF7, AF8, FPz, FP1, FP2) were not included to minimize the impact of ocular artifacts (Acharya et al., 2016). Iz was excluded from re-referencing as it was found to be noisy in a subset of recordings.

The use of high-pass filtering is disputed in the ERP literature (Rousselet, 2012; Tanner et al., 2015); however, 0.1 Hz is reported as the cutoff frequency with an optimal trade-off between waveform distortion and noise reduction (Acunzo et al., 2012). In this work, because the Biosemi recording system keeps the DC component of the signal, the data were high-pass filtered at 0.1 Hz using a 1st-order Infinite Impulse Response filter, and forward-backward filtered using the filtfilt() function to avoid onset distortion (Rousselet, 2012). A second-order low-pass Butterworth filter with a cutoff frequency of 20 Hz was then applied to remove powerline noise and other high-frequency artifacts, such as muscular, ocular or ECG (Rousselet, 2012).

Data was segmented into epochs, from 200 ms before stimulus onset to 1 s after the stimulus end. Although a filter was used, removing slow drifts and baseline DC offset correction was still required to eliminate artefactual effects introduced by the filter itself (Tanner et al., 2016). A typical baseline correction was subsequently applied based on the mean of the 200 ms pre-stimulus period.

To investigate the acute effects of VNS, each stimulus of the VNS ON condition was labeled as VNS ON HIGH or VNS ON LOW based on its precise temporal location within the VNS duty cycle. Epochs from the VNS ON condition were labeled VNS ON HIGH when the auditory stimuli occurred during the high intensity plateau (labeled 1 in Figure 1), and VNS ON LOW when they occurred during the OFF portion of the duty cycle (labeled 3 in Figure 1). Epochs falling within the ramp-up or ramp-down transitions (labeled 2 in Figure 1) were excluded from further analyses but were included in the VNS ON analysis. The temporal structure of the duty cycle was inferred from the VNS signal recorded through additional external electrodes.

Finally, epoch rejection was performed by discarding epochs with an amplitude exceeding ±75 μV or lacking a patient’s motor response. Moreover, a 100 ms sliding window with a 50 ms step size was applied to the HEOG signal to exclude EEG epochs with HEOG fluctuations > 16 μV.

Mean waveforms were computed separately for target and non-target epochs before being subtracted to create the ERP differential wave. The P300 was then identified on the Pz electrode as the largest peak within the 230–650 ms post-stimulus window. Peak detection was manually verified across all conditions and patients to ensure accurate distinction between P3a and P3b. The metric selection can bias P300 amplitude and latency estimates (Luck, 2014). Different methods were implemented and are provided in Supplementary material 2. For consistency with the methodology employed in P300 VNS literature (Neuhaus et al., 2007; De Taeye et al., 2014; Hödl et al., 2020), the presented results are based on the amplitude and latency of the highest detected peak. Additional measures include reaction time and response accuracy, respectively defined as the time taken by the participant to press the button and the number of targets detected.

### 2.5 Statistical analysis

Demographic differences between responders and non-responders, including sex, age, epilepsy duration, number of ASMs, and VNS duration, were investigated using Fisher’s exact test and Mann–Whitney’s test.

Linear mixed-effects models (LMMs) were used to assess the relationship between experimental conditions and behavioral performances or cognitive responses. Each model predicted accuracy, reaction time, amplitude or latency from the interaction between VNS response (responders, non-responders) and experimental conditions (VNS OFF, VNS ON, VNS ON HIGH, VNS ON LOW), while participants were modeled as random intercepts with a fixed slope configuration. Both predictors (response and condition) were treated as categorical variables. As established in standard LMM methodology (McCulloch and Searle, 2000), in the primary model specification, responders served as the reference for response, and the VNS OFF condition served as the reference level for condition. Pairwise contrasts of interest were obtained by re-leveling the categorical predictors, without refitting the model, and are reported in Supplementary material 1. To assess significance or the main effect and interactions, Type III ANOVA with Satterthwaite’s approximation was applied, to ensure correct inference for main effects and interactions (Luke, 2017).

The relationship between the modulatory effects of VNS and the degree of responsiveness was assessed using Spearman’s correlation between the CRRS and the differential measures of amplitude and latency, computed as changes from VNS OFF to VNS ON (*Δ* amplitude and Δ latency, respectively).

Finally, given that in the P3b ERP literature, amplitude modulation is often associated with latency modulation (Polich et al., 1997; Polich, 2007; Demirayak et al., 2023), a simple classification framework was implemented using the ratio of amplitude to latency. This index was computed separately for the VNS OFF and VNS ON conditions. For each participant, the change in this index between conditions (Δindex = ΔON - ΔOFF) was used as the classification feature. Given that the primary objective was to provide a proof-of-concept illustration rather than to develop or optimize a predictive model, no training phase or parameter estimation was performed. Instead, a simple rule-based classifier was defined *a priori*, based on the direction of the modulation. Specifically, a decrease in the amplitude-to-latency ratio from VNS OFF to VNS ON (Δindex < 0) was interpreted as indicative of responder status, whereas an increase (Δindex > 0) was associated with non-responder status. As no training phase or parameter estimation was performed, the classification rule was directly applied to the full cohort, and performance was evaluated using the resulting confusion matrix, reporting true positives (TP), true negatives (TN), false positives, and false negatives (FN), along with accuracy (TP + TN)/ (TP + TN + FP + FN), sensitivity (TP/TP + FN), and specificity TN/(TN + FP) were derived. This composite P3b index was thus used to investigate whether differential VNS-induced effects could efficiently discriminate responders from non-responders. All statistical effects were evaluated at the 0.05 significance level, and all analyses were conducted in MATLAB.

## 3 Results

### 3.1 Clinical and demographic information

The demographic distribution is summarized in Table 2, which includes median values, Inter-Quartile Range, and resulting statistics, along with their corresponding *p*-values. No significant differences were identified in the demographic data, suggesting that the two groups have similar demographic and clinical profiles. However, among the clinical parameters, a significant difference was observed in the number of ASMs used between the two groups, with a higher number in non-responders.

**Table 2:** Between-group comparison of demographic and clinical characteristics.

Characteristics	Response group R (*n* = 7)	Response group NR (*n* = 8)	*p*-value
Sex	Male 3	Male 5	0.6193 (F)
	Female 4	Female 3	
Age [years], Median, (IQR)	33 (27.5–37.5)	43.5 (32.5–49)	0.221 (M-W)
Number of ASMs, Median, (IQR)	2 (2–2)	3 (3–3)	**0.013 (M-W)** ^*^
Epilepsy duration [years], Median, (IQR)	22 (16–32.5)	22 (15–35.5)	0.866 (M-W)
VNS follow-up [years], Median, (IQR)	6.5 (3.25–9.75)	2 (2–6.75)	0.189 (M-W)

Data is provided as median (Inter-Quartile Range). (F): Fisher’s exact test; (M-W): Mann–Whitney U test. Significant *p*-values were highlighted in bold.

### 3.2 Effect of VNS modulation on behavioral response

The results of the LMM analyses investigating the impact of VNS on patient behavioral response are reported in Table 3, along with mean values for each group across experimental conditions. Comprehensive LMM statistics, including all associated p-values, are reported in Supplementary material 1.

**Table 3:** Groups’ behavioral and electrophysiological responses and LMM analysis results.

Behavioral results						LMM statistics		
		VNS OFF	VNS ON	VNS ON LOW	VNS ON HIGH	Condition	Response	Interaction
Accuracy [%]	R	97.960 ± 1.642	96.233 ± 6.243	95.483 ± 4.228	96.865 ± 7.252	*p* = 0.139	*p* = 0.550	*p* = 0.975
	NR	98.513 ± 1.771	97.260 ± 3.063	96.146 ± 1.86	98.016 ± 5.250			
Reaction time [s]	R	0.430 ± 0.098	0.475 ± 0.100	0.477 ± 0.116	0.471 ± 0.091	*p* = 0.008**	p = 0.975	*p* = 0.363
	NR	0.405 ± 0.056	0.420 ± 0.047	0.422 ± 0.047	0.419 ± 0.049			
Electrophysiological results								
Peak amplitude [μV]	R	7.467 ± 5.515	5.787 ± 4.689	6.604 ± 5.427	6.064 ± 4.251	*p* = 0.475	*p* = 0.121	*p* < 0.001***
	NR	8.525 ± 3.246	10.324 ± 4.249	10.619 ± 4.775	10.534 ± 4.272			
Peak latency [s]	R	0.402 ± 0.063	0.484 ± 0.133	0.494 ± 0.098	0.501 ± 0.090	*p* = 0.285	*p* = 0.056	*p* < 0.001***
	NR	0.432 ± 0.096	0.384 ± 0.054	0.373 ± 0.064	0.403 ± 0.062			

Mean values and standard deviation of behavioral and electrophysiological results for the VNS OFF, VNS ON, VNS ON LOW, and VNS ON HIGH conditions. Results from the Type III ANOVA with Satterthwaite’s approximation applied to the linear mixed model are reported for the main effects of condition, response group, and their interaction. *p*-values are presented after FDR correction, along with *p*-values when significance was reached before correction. The level of statistical significance was set at *p* < 0.05 and indicated with ^*^, the level of *p* < 0.01 with ^**^ and *p* < 0.001 with ^***^. Significant *p*-values were highlighted in bold.

**Figure 2:**
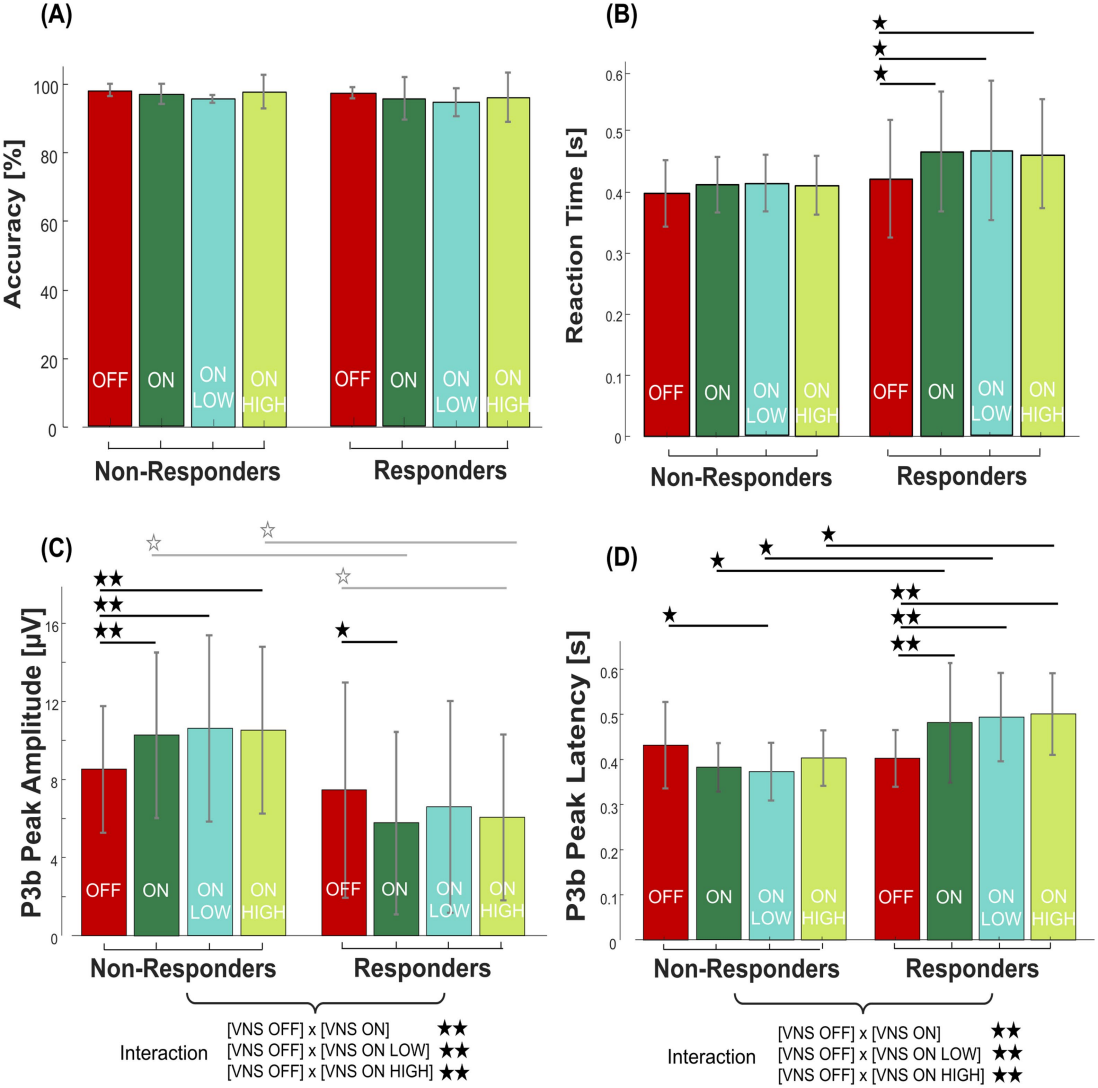
Behavioral and electrophysiological responses of non-responders and responders including accuracy **(A)**, reaction time **(B)**, P3b peak amplitude **(C)**, and P3b peak latency **(D)**. Mean values and standard deviation for each group are shown for all experimental conditions and measures. Filled black stars indicate effects that remained significant after FDR correction in the linear mixed model analysis, whereas empty grey stars denote significant effects before correction. Statistical significance levels are marked with one star for *p* ≤ 0.05, two stars for *p* ≤ 0.01, and three stars for *p* ≤ 0.001.

The analysis revealed a significant main effect of condition on reaction time [*p* = 0.008], suggesting that at least one experimental condition exerted a significant differential influence on behavioral performance. Post-hoc comparisons indicated that the observed effect was primarily driven by a reduction in performance among responders only during VNS stimulation, as illustrated in Figure 2B for response time. This reduction was characterized in responders by a significant increase in reaction time when VNS was enabled in the VNS ON [p_fdr = 0.024], VNS ON LOW [p_fdr = 0.016], and VNS ON HIGH [p_fdr = 0.037] as compared to VNS OFF condition. However, as the interaction between condition and response did not reach statistical significance, this suggests that during this experiment, the effect of VNS on behavioral performance was not differentially modulated by group status and was restricted to responders. No significant differences were observed for accuracy.

### 3.3 Effect of VNS modulation on P3b electrophysiological response

The results of the LMM investigating the P3b response are also presented in Table 3, showing the mean and standard deviation of peak amplitude and latency for each group across all experimental conditions. Detailed individual data and waveforms are provided in Supplementary material 1.

Analyses of the impact of VNS on P3b ERP show a strongly significant effect of the interaction between the experimental conditions and the response group for both amplitude [p_fdr < 0.001] and latency [p_fdr < 0.001]. Subsequent post-hoc analysis, illustrated in Figure 2C for P3b peak amplitude and Figure 2D for peak latency, revealed that for amplitude, the interaction effect was present between the two groups when comparing VNS OFF with VNS ON [p_fdr = 0.002], VNS ON LOW [p_fdr = 0.004], and VNS ON HIGH [p_fdr = 0.002]. More specifically, in the non-responders, the P3b amplitude was increased across all conditions where VNS was enabled, respectively, VNS ON [p_fdr = 0.009], VNS ON LOW [p_fdr = 0.004], and VNS ON HIGH [p_fdr = 0.004], as compared to VNS OFF. In contrast, a significantly smaller amplitude was observed in responders during VNS ON [p_fdr = 0.023] and a trend in VNS ON HIGH [*p* = 0.059]. In addition, trends in group differences can be observed under VNS ON [p_fdr = 0.076] and VNS ON HIGH [p_fdr = 0.076], where, on average, non-responders exhibit a higher amplitude than responders.

For latency, the interaction effect was significant between the two groups between VNS OFF and VNS ON [p_fdr = 0.003], VNS ON LOW [p_fdr = 0.001], and VNS ON HIGH [p_fdr = 0.003]. Within-groups, these differences were reflected in non-responders with a smaller P3b latency trend during VNS ON [*p* = 0.078] and a significant difference during VNS ON LOW [p_fdr = 0.032] compared to VNS OFF. In responders, the opposite effect was visible with a higher latency across all conditions where VNS was enabled: VNS ON [p_fdr = 0.007], VNS ON LOW [p_fdr = 0.003], and VNS ON HIGH [p_fdr = 0.003] as compared to VNS OFF. Between-group comparison revealed that on average, the non-responder group showed a smaller latency than responders when VNS was enabled in VNS ON [p_fdr = 0.032], VNS ON LOW [p_fdr = 0.011], and VNS ON HIGH [p_fdr = 0.033].

Taken together, these findings suggest that VNS stimulation, i.e., the VNS ON condition, elicited group-specific effects on P3b characteristics, with responders exhibiting lower amplitudes and prolonged latencies, whereas non-responders demonstrated the opposite pattern.

### 3.4 Direct effect of VNS pulses on P3b behavioral and electrophysiological response

VNS stimulation pulses did not acutely modulate behavioral and electrophysiological responses. Indeed, post-hoc analyses, illustrated in Figure 2, showed no significant interaction between VNS ON LOW and VNS ON HIGH and the groups of response for either accuracy, reaction time, P3b amplitude, or P3b latency. Similarly, within-groups comparisons indicated no significant differences between VNS ON LOW and VNS ON HIGH in either non-responders or responders across all measures, suggesting that VNS stimulation pulses did not elicit differential effects from stimulation breaks. Between-groups differences, already reported in the previous sections, show patterns similar to those observed under the general VNS ON condition, without evidence of enhanced statistical power.

Overall, these findings indicate that VNS activation elicits an acute response on P3b characteristics (i.e., VNS ON condition significantly differed from VNS OFF), but no direct pulse-locked modulation (i.e., VNS ON HIGH condition did not differ significantly from VNS ON LOW). Nevertheless, to discriminate between the two groups of responses, VNS ON HIGH showed greater consistency, with trends and significant differences observed for amplitude and latency, respectively. VNS ON LOW showed differences between the two groups only for latency, but with a higher degree of significance.

It should be noted that, at the individual level, the mean of the VNS ON HIGH and VNS ON LOW subsets is not expected to fall within that of the overall VNS ON condition. This is because (i) ramp-up and ramp-down epochs are included in VNS ON but excluded from both subsets, such that the VNS ON condition is not a strict combination of VNS ON HIGH and VNS ON LOW; (ii) the number of contributing epochs may differ across subsets due to the pseudo-random task structure; and (iii) peak-based ERP metrics are non-linear measures derived after averaging, and are therefore not constrained by subgroup means.

### 3.5 Correlation of the clinical research response scale with P3b response

**Figure 3:**
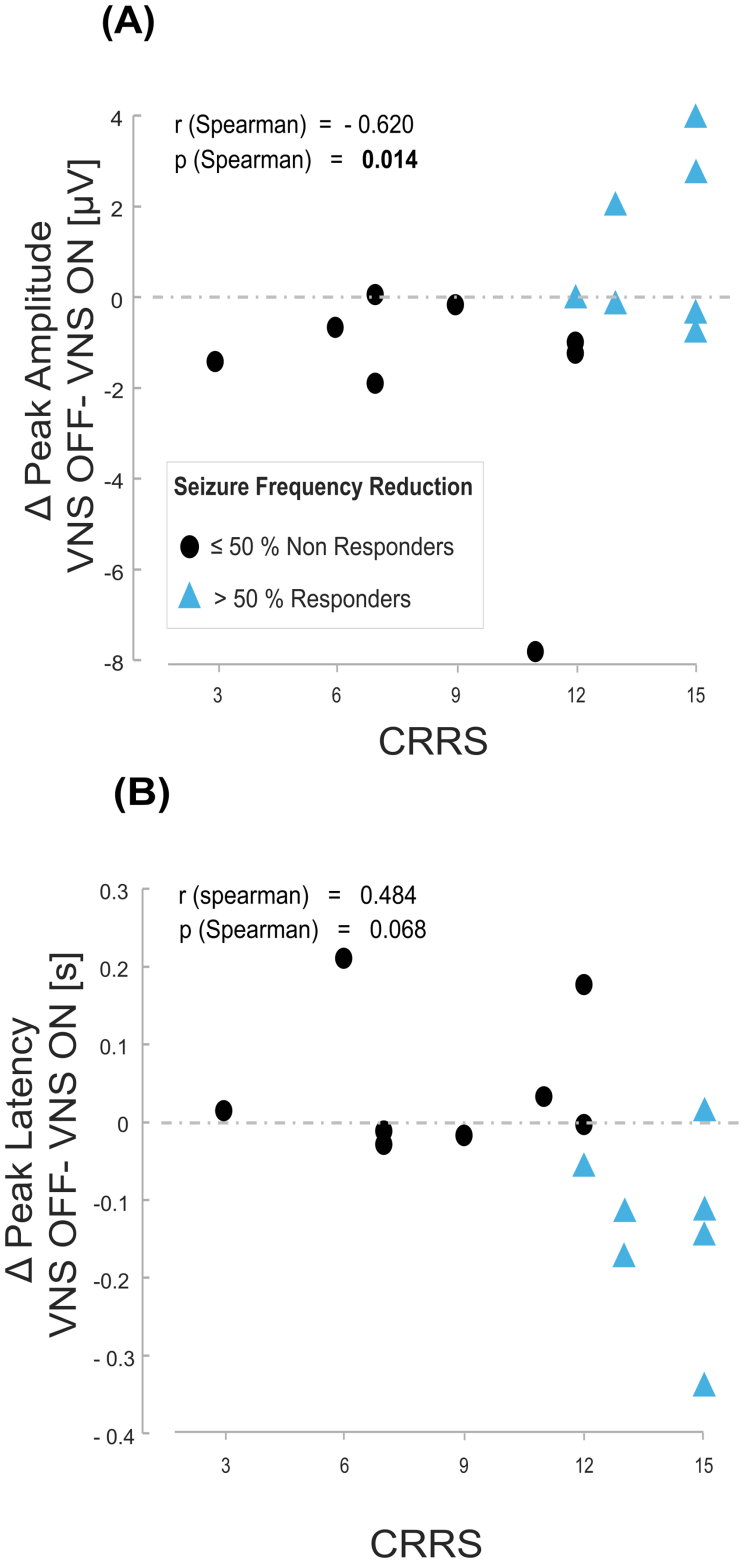
Correlation of the Clinical Research Response Scale (CRRS) and the VNS-induced modulation of P3b amplitude (top panel) and latency (bottom panel). For each participant (*n* = 15), the difference between VNS OFF and VNS ON conditions is plotted. Dots indicate individuals classified as non-responders and triangles responders following a clinical binary classification criterion (≤ 50% seizure frequency reduction associated with non-responders). Spearman coefficients and associated *p*-values are indicated, with a significant association observed for amplitude (*p* = 0.014).

The results of the correlation analysis are reported in Figure 3. In Figure 3A, the *Δ* amplitude exhibited a near-significant positive correlation with the CRRS (r_spear = 0.484, p_spear = 0.068), indicating an increase in Δ amplitude with higher CRRS scores. Consistently, in Figure 3B, the Δ latency was negatively correlated with the score (r_spear = − 0.620, p_spear = 0.014*), reflecting a decrease in Δ latency with increasing score.

### 3.6 Composite index of P3b characteristics as a VNS response classification rule

**Figure 4:**
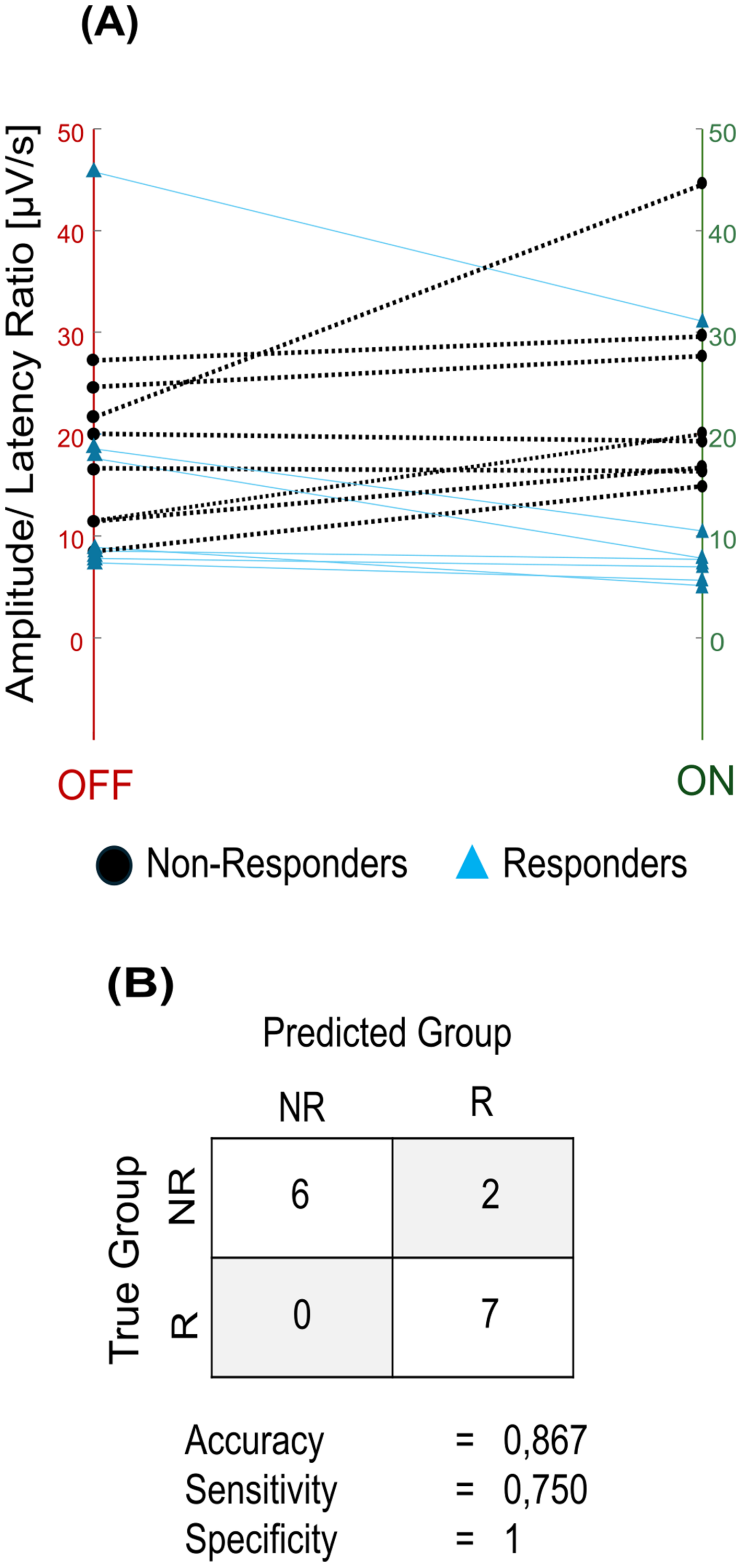
Classification of non-responders and responders based on the VNS-induced modulation of P3b characteristics. **(A)** A composite P3b index, defined as the amplitude-to-latency, was computed for VNS OFF and VNS ON conditions. The modulatory impact of VNS on P3b results in an increase of the index in non-responders (≤ 50% seizure frequency reduction) and a decrease in responders (> 50% seizure frequency reduction). **(B)** A classification framework based on the sign of the index difference between VNS OFF and ON was applied on the experimental cohort as a proof-of-concept, resulting in an overall accuracy of 86.7%, correctly identifying 75% of non-responders and 100% of responders.

The composite index of P3b amplitude and latency was evaluated across the VNS OFF and ON conditions, with the direction of the change reflecting performance variation, as illustrated in Figure 4A. Given the results of the LMM analysis, an increase in the ratio, reflected in a positive slope from VNS OFF to VNS ON, would be indicative of non-responder status, whereas a negative slope would be associated with responders. The classification analysis, based on the sign of the change in the amplitude-to-latency ratio between the VNS OFF and VNS ON conditions, yielded a global accuracy of 86.7%, with 13 out of 15 patients correctly identified. In terms of class-specific performance, the method achieved a sensitivity of 75% (6 out of 8 non-responders correctly classified) and a specificity of 100% (all 7 responders correctly identified). These results are illustrated in Figure 4B.

## 4 Discussion

Given the anatomical projection of the vagus nerve to the LC-NE system, modulation of vagal activity through VNS may exert an indirect influence on LC neuronal activity (Groves et al., 2005; Sara, 2009; Thompson et al., 2019). The present study sought to advance the understanding of individual variability in VNS response by examining the impact of this therapy on LC-NE through P3b. Furthermore, we investigated the direct impact of stimulation pulses and the evolution of this effect across a response scale, the CRRS (Danthine et al., 2025). Ultimately, assessing whether such modulation can serve as a marker of therapeutic efficacy.

Our findings show a group-specific and opposite acute effect of VNS. Indeed, we found a significant interaction between stimulation condition and clinical response (Table 3). VNS was associated with reduced P3b amplitude and prolonged latency in responders, whereas non-responders showed the opposite pattern. These opposite effects produced trends and significant between-group differences in P3b amplitude and latency when the device was enabled. At the individual level, the magnitude of VNS-induced changes in P3b amplitude and latency was significantly associated with the CRRS, showing a monotonic trend, meaning that patients with higher scores showed consistently larger changes. When combined into a composite index reflecting the overall P3b modulation, it enabled a preliminary discrimination between responders and non-responders with an accuracy of 86.7%.

Finally, these findings provide an initial insight into the timing of VNS effects. Indeed, VNS activation could elicit an acute response (i.e., VNS ON condition significantly differed from VNS OFF), but we could not observe a direct pulse-locked modulation (i.e., VNS ON HIGH condition did not differ significantly from VNS ON LOW within either group).

### 4.1 Group-specific and opposite effects of VNS on LC-NE

The results obtained suggest a VNS-induced activation of the LC-NE system, as evidenced by modulation of P3b amplitude and latency, specific to therapeutic response. Notably, P3b latency showed a consistent pattern with amplitude: higher amplitude was associated with shorter latencies, and lower amplitude with longer latencies, as commonly reported in the literature (Polich et al., 1997; Polich, 2007; Demirayak et al., 2023). Although the effects of VNS on P3b latency have not been reported as significant in the DRE literature (De Taeye et al., 2014; Hödl et al., 2020), P3b latency is also a robust marker of ERP modulation, as it reflects the timing of the neural response and is stable across replications (Segalowitz and Barnes, 1993; Walhovd and Fjell, 2002; Perez et al., 2016). This difference from other studies could be attributed to the methodology employed. Indeed, substantial latency jitter across trials can make peak-based latency estimates within the 250-ms to 600-ms post-stimulus timeframe less representative of P3b true timing (Luck, 2014). In our work, multiple metrics were implemented within the same LMM (see Supplementary material 2), and all showed a significant interaction effect, confirming that VNS influences P3b differently in responders and non-responders.

The VNS-induced acute effect is evidenced by the absence of significant differences between the two groups under the VNS OFF condition. The absence of significant differences when VNS is disabled is consistent with previous literature, which found no difference between responders and non-responders in a similar population (DRE patients who benefited from VNS therapy over 18 months) (De Taeye et al., 2014). This observation was also reported by (Neuhaus et al., 2007). However, they targeted patients living with major depressive disorders (MDD). Still, as both epilepsy and MDD might result from the same underlying pathological lack of noradrenergic signaling (Jobe, 2003), these results could be extended to people living with epilepsy. Additional works investigating the relationship between auditory P3b and VNS include those by Brázdil et al. (2001) and Hammond et al. (1992), which found no significant impact. However, both studies had relatively small sample sizes and, unlike our work, did not differentiate results between responders and non-responders.

The modulatory effect of VNS on P3b observed in this study differs from the findings of De Taeye et al. (2014), in that we find VNS to be associated with reduced performance in responders. Moreover, we observed an effect of VNS on non-responders, which has not yet been reported in the literature. We believe that methodological differences may account for these differences. First, our artefact rejection process included the exclusion of frontal electrodes from the average reference, whereas De Taeye et al. (2014) used independent component analysis. This distinction may have influenced the measurement of P3b at Pz, potentially rendering it more similar to a signal recorded at Cz and thereby capturing a more frontal aspect of P300.

Second, in De Taeye et al. (2014), a duty cycle of 7 s ON and 18 s OFF was applied. In contrast, our experimental protocol used a longer ON phase of 14 s with the same OFF phase of 18 s. Animal studies have shown that longer VNS pulse-train duration (i.e., longer the ON phase of the duty cycle) leads to a linear increase in LC-NE neuron-driven spikes (Hulsey et al., 2017). Although LC-NE neurons appear to be linearly driven by electrical stimulation, this does not imply that the associated cognitive function of arousal exhibits the same linearity. Existing models suggest an inverted U-shaped relationship between LC-NE activity and arousal (Aston-Jones et al., 1999). Indeed, LC-NE neurons exhibit two modes of activation, phasic and tonic, respectively characterized by brief bursts of action potentials and baseline state-dependent firing patterns, typically oscillating between 0.1 and 5 Hz (Aston-Jones and Bloom, 1981). Models hint at a synergy between the two modes, modeled by analogy to the Yerkes-Dodson curve, in which an optimal level of tonic activity, coupled with bursts of phasic activity, yields better performance. In contrast, lower and higher levels of tonic activity were associated with poorer performance on discriminatory tasks (Aston-Jones et al., 1997). VNS impacts both modes of activation. Extensive rodent literature demonstrates that both acute and chronic VNS increase the firing rate of LC neurons, reflecting a direct modulation of phasic activity (Groves et al., 2005; Dorr and Debonnel, 2006). Beyond these phasic effects, VNS also alters tonic activity. For instance, a higher number of bursts per minute was reported after 14 days of VNS implantation in rats (Manta et al., 2009a), and increases in the basal firing rates of LC neurons have been observed after both short- and long-term VNS (Dorr and Debonnel, 2006). In cognitive and behavioral studies, P3b is considered an electrophysiological correlate of the LC-NE phasic response, where the amplitude and latency of P3b, respectively, denote the intensity and timing of discharge [see for review in Nieuwenhuis et al. (2005)]. A higher amplitude suggests a greater modulatory impact of VNS on the phasic activity of LC-NE neurons.

This interpretation is further supported by the results yielded by our simple rule-based classification analysis. Effective engagement of the LC-NE by VNS is expected to manifest as an increase in P3b amplitude and a shorter latency during VNS ON relative to VNS OFF. In our model, this is reflected in a positive Δindex value for the non-responder group. Conversely, saturation would be associated with a smaller amplitude and a larger latency under VNS ON, leading to a negative Δindex, which is associated here with responders. All individuals clinically identified as responders were also classified as such by our *a priori* classification rule, yielding a specificity of 100%. Among non-responders, seven were correctly identified, and two were misclassified as responders. Although this approach does not yet enable prediction of pre-implantation responses, it already provides an objective indicator of therapeutic engagement. Such a marker could support parameter titration in responders and facilitate the early identification of non-responders who may benefit from alternative therapeutic strategies. Notably, despite the absence of parameter learning, our rule achieved a specificity of 100% and a sensitivity of 75%, which is comparable to previous attempts using P300-based criteria (specificity 90%, sensitivity 75%) (De Taeye et al., 2014). In addition to the LC-NE-based approach, several groups have explored classifying VNS responders and non-responders using machine-learning models. These include a predictive framework based on thalamocortical microstructural features (accuracy of 85–90%) (Berger et al., 2024b) and, in pediatric populations, connectomics and entropy-driven metrics (accuracy of 80–85% with AUC values >0.85) (Cheng et al., 2024)scalp-EEG functional connectivity patterns (~82% with an AUC of 0.86) (Chen et al., 2023), and synchronization biomarkers integrated with clinical variables (~85–90% accuracy) (Ma et al., 2022).

We believe that, through its impact on vagal afferences, VNS modulates the state of the brains of patients in different states, leading to distinct P3b responses. These differences may stem from the fact that responders exhibit greater sensitivity to VNS both in the short- and long-term. From the literature, we know that, before implantation, responders may exhibit lower LC activity, resulting in a smaller P3b amplitude than non-responders (Hödl et al., 2020). However, chronic effects modulate this activity, increasing it to a level similar to that of non-responders, as suggested by the lack of differences between groups after therapy when VNS is disabled (Neuhaus et al., 2007; De Taeye et al., 2014). The acute effects of VNS are reflected by a higher P3b amplitude in VNS responders when the device is enabled (De Taeye et al., 2014). These findings suggest that responders exhibit sensitivity to VNS modulation of LC activity, consistent with the antiepileptic role of LC release in reducing seizure susceptibility and severity (Berger et al., 2024a). We believe that the absence in the literature of significant amplitude or latency changes in non-responders, both when VNS is disabled and following therapy, may indicate lower sensitivity rather than an absence of effect. This hypothetical difference in sensitivity between responders and non-responders is supported by our findings, which show that modulation in amplitude and latency is correlated with response score. Indeed, a larger difference, hence a greater modulatory impact of VNS, is associated with a higher score. Findings in the literature are consistent with our observations, as modulation in P3b amplitude was correlated with VNS response pre- and post-therapy in epilepsy (Brázdil et al., 2001) and MDD (Neuhaus et al., 2007). Taken together, a high dose of LC-NE stimulation might lead to different outcomes in responders compared to non-responders, as suggested by the significant interaction effects observed here between groups and the ON and OFF conditions. We believe that by increasing the dose through longer stimulation pulse trains, we could have saturated the LC-NE systems, leading to hyperexcitability and a decrease in cognitive performance, whereas the same dose impacted non-responders less, resulting in a global increase in performance.

### 4.2 Impact of stimulation pulses

This study represents the first characterization of the importance of VNS timing dynamics. The observed effects manifest on a timescale of minutes. Indeed, they are detected under the VNS ON condition, but are absent, hence disappear under VNS OFF. However, the observed effects do not manifest on a timescale of seconds. Indeed, VNS ON HIGH and the VNS ON LOW conditions do not differ, hinting that the effects of the electrical pulses persist under VNS ON LOW and are reflected during the 18 s of VNS ON LOW.

To elucidate the underlying mechanism with greater precision, it would be necessary to adjust the duration of the duty cycle during VNS ON LOW. Extending the breaks between stimulation trains would allow the evaluation of whether longer intervals can reveal the acute effects of VNS without the saturation observed here.

While these results provide important insights into the timing of VNS effects, their mechanistic interpretation remains limited by the simplified representation of the underlying neuromodulatory pathways (i.e., interaction between VNS and LC-NE). In addition to the LC, the vagus nerve also anatomically activates the dorsal raphe and basal forebrain, which release serotonin and acetylcholine, respectively (Manta et al., 2009b; Bowles et al., 2022; Martin et al., 2024). Broad modulation of afferent pathways of the central nervous system is likely to mediate the effect of VNS, making it difficult to isolate the role of one pathway from another. In addition, in this work, the activity of the P3b is thought to reflect the activity of the LC-NE; however, as with VNS, numerous cortical generators are involved in the course of P3b (Volpe et al., 2007; Wronka et al., 2012).

### 4.3 Limitations and future perspectives

This work presents several limitations. Cofactors could not be added as additional explanatory variables due to the small sample size. Although benefiting from limited ASMs improvement, our DRE patients still undergo drug treatment. Previous studies have shown that commonly used antiepileptic medications can affect P3b: Carbamazepine and Topiramate tend to reduce amplitude and prolong latencies (Shafiyev and Karadaş, 2024), whereas others, including levetiracetam, may enhance cognitive performances, impacting P3b amplitude (Gongora et al., 2021). Furthermore, polytherapy (two or more ASMs) has been associated with reduced amplitude and prolonged latency relative to monotherapy (Saric Juric et al., 2021). However, in this study, despite the challenges in discerning the impact of medication from that of VNS, no differences were found between the groups under VNS OFF. Additional cofactors include the duration of epilepsy. Indeed, age at onset and duration of epilepsy are, respectively, positively and negatively correlated with P3b amplitude, and negatively and positively correlated with P3b latency (Hasan et al., 2023). Finally, both latency and amplitude are impacted by the type of epileptic seizure (Zhong et al., 2019). Even if a demographic analysis revealed no significant differences between the two groups, further analysis would require including ASMs, age at onset, duration of epilepsy, and seizure type as explanatory variables in the LMM model to reflect the complex interactions among these variables. This, however, would require a larger sample size.

Further work should explore the dose–response relationship between the VNS duty cycle and LC-NE modulation. Indeed, the use of an ON duration longer than 14 s, as in our study, should lead to poorer performance in non-responders and better performance in responders. In contrast, reducing the duration of the ON phase to 7 s would improve performance only in responders, as observed by (De Taeye et al., 2014).

Although no significant differences were observed between the groups when VNS was OFF, our study, along with the literature, suggests that responders have a significantly higher sensitivity to VNS than non-responders. It is therefore essential to investigate how the effect of VNS evolves following implantation. If an increase in performance compared to before therapy is observed only in responders, similar to the work of Neuhaus et al. (2007), it would support the hypothesis that this sensitivity supports cumulative therapeutic effects.

Finally, the results of our classification should be interpreted with caution, as they arise from a simple, non-optimized, rule-based approach applied to the entire dataset. As such, they do not reflect the performances of a validated predictive model but rather provide an encouraging proof-of-concept supporting the discriminative potential of P3b characteristics. To go further, future studies will need to implement a fully developed predictive modeling framework. This would require the construction of richer multivariate feature vectors, the use of classifiers with parameter learning, and a clear separation between training and test data sets, which would require a larger cohort. In this framework, appropriate validation strategies, such as cross-validation, are essential for assessing model generalizability. This model would help translate the observed electrophysiological modulation observed in our work into a robust, clinically meaningful rule for differentiating clinical response groups.

## Data Availability

The raw data supporting the conclusions of this article will be made available by the authors, without undue reservation.
