# Supplementary material for: Vagus Nerve Stimulation differentially modulates P3b in responders and non-responders: toward a biomarker of therapeutic efficacy
Source: Front Neurosci. 2026 Jun 17;20:1786262. doi: 10.3389/fnins.2026.1786262 (PMC13320354; doi:10.3389/fnins.2026.1786262)
Supplement: Supplementary file 1 [file Data_Sheet_1.DOCX]

Supplementary Material - 1

# Supplementary Data

**Detailed Individual Response Score, Behavioral and Electrophysiological Results and Statistical Analysis**

These supplementary materials thoroughly develop the data presented in the main body of the manuscript.

First, the Clinical Research Response Scale (CRRS) framework, developed in the work of Danthine et al. [1], is presented including the question grid (Supplementary Table 1), which contains all questions asked to the participants along the associated score. First, the impact of the magnet (patient controller) is assessed, and if the percentage of seizure interrupted by its use is above 50%, +5 points are given. If the number of is below 50% but non-null, +4 points are given and if in addition any of the following benefit is met: diminution of seizure duration, seizure intensity and post-ictal remission duration, +1 can be given. If the magnet does not interrupt any crisis, any following benefit gives a +1 each: diminution of seizure duration, seizure intensity and post-ictal remission duration, +1 can be given. In addition, the impact of VNS was assed more globally, by first determining the seizure frequency reduction, computed as the mean of the reduction for each type of seizure, with the baseline taken as the mean seizure frequency 1 year before VNS implantation and the mean seizure frequency 1 year before the experiment. If the reduction was above 80%, +10 were added, between 50% and 30% the score was gradually adapted, ranging from +8 to +3 and if any of the following benefits were observed: diminution of seizure duration, seizure intensity and post-ictal remission duration and additional +1 was given. If the reduction was below 30% any of the benefits (diminution of seizure duration, seizure intensity and post-ictal remission duration), could add an additional +1 each. The finial score is computed as the sum of both benefit from the use of patient controller and VNS. For each individual, the score is indicated in Supplementary Table 2, along the binary scoring (R >50% or NR ≤50% seizure reduction assed under the same timeframes).

Secondly, the individual results are presented for each participant. Supplementary Figure 1 presents the ERP waveforms for each experimental condition (VNS OFF, VNS ON, VNS ON HIGH, VNS ON LOW), with the detected P3b. The behavioral results for mean accuracy and reaction time are presented in Supplementary Table 3 for each individual across all conditions. The exhaustive values of the electrophysiological results, including measured P3b peak amplitudes and latencies, are presented for every participant and condition in Supplementary Table 4.

Finally, the results of the LMMs, including the model coefficient estimates with SE, t-statistics, confidence intervals, p-values, and p-values corrected using FDR. Results of the models using the following metrics as dependent variable: reaction time, accuracy, P3b peak amplitude, P3b peak latency are respectively shown in Supplementary Table 5, 6, 7, 8. For each model, the group of reference is responders, and the condition of reference is indicated in brackets.

# Supplementary Figures and Tables

## Supplementary Figures


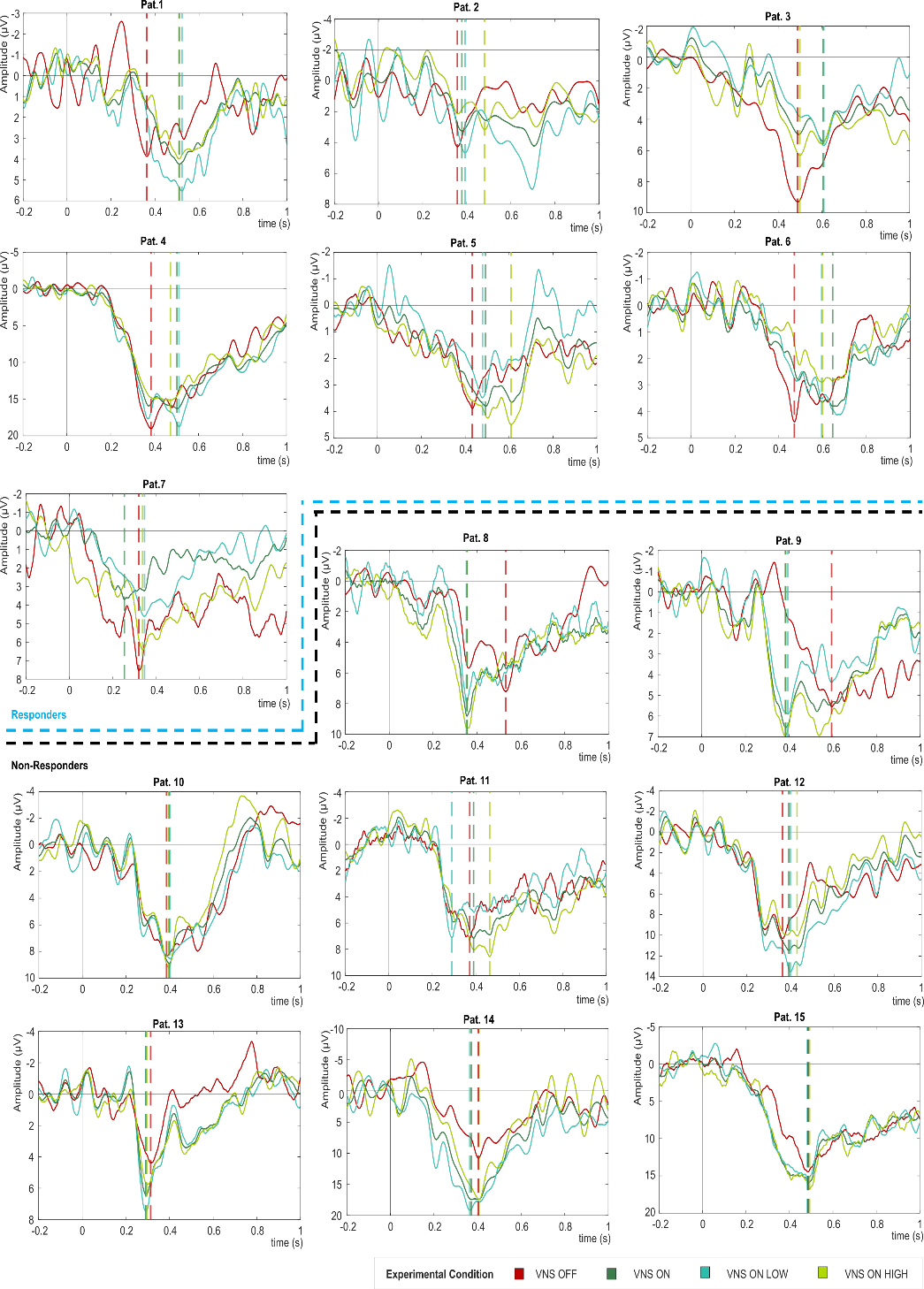


**Supplementary Figure 1.** Differential ERP waveforms (between target and non-target) across all individual participants and conditions. Responders are plotted on the left and responders on the right, for VNS OFF, VNS ON, VNS ON LOW, and VNS ON HIGH respectively in red, green, turquoise, and light green. For each participant and condition, the P3b is highlighted as the highest peak within the 230 ms to 650 ms window with a dashed line.

## Supplementary Tables

**Supplementary Table 1**. Methodology applied to score the patient response to VNS according to the Clinical Research Response Scale (CRRS), developed in the work of Danthine et al. [1]. The patients had to evaluate how the use of their magnet impacted their seizure frequency, which was scored accordingly (>50% = +5, ≤50% = +4, 0% = 0). The impact of the magnet on seizure duration, intensity, and post-ictal duration was also described, and could score +1 if the seizure frequency was less than 50%. Chronic response was evaluated by first estimating the monthly seizure frequency at the time of the experiment and then comparing it to the monthly seizure frequency before VNS implantation. Impact of VNS on seizure duration and intensity and post-ictal duration was assessed and could contribute to the overall score if the seizure frequency was < 80%.

| **Response Type** | **VNS Effect** | **Criterion** | **Score Computation** | | | | **Total Score** |
| --- | --- | --- | --- | --- | --- | --- | --- |
| **Acute – Magnet Response** | | | | | | | **0-5** |
|  | Seizure-suppressing effect of the magnet | >50% | +5 | | | |  |
|  |  | <50% | +4 | Seizure duration ↘  Seizure intensity ↘  Post-ictal duration ↘ | Yes / No  Yes / No  Yes / No | If at least one “yes”: +1 |  |
|  |  | 0% | +0 | Seizure duration ↘ | Yes / No | If “yes”: +1 |  |
|  |  |  |  | Seizure intensity ↘ | Yes / No | If “yes”: +1 |  |
|  |  |  |  | Post-ictal duration ↘ | Yes / No | If “yes”: +1 |  |
| **Chronic** | | | | | | | **0-10** |
|  | Seizure frequency reduction | >80% | +10 | | | |  |
|  |  | >50% | +8 | Seizure duration ↘  Seizure intensity ↘  Post-ictal duration ↘ | Yes / No  Yes / No  Yes / No | If at least one “yes”: +1 |  |
|  |  | =50% | +7 |  |  |  |  |
|  |  | >30% | +6 |  |  |  |  |
|  |  | =30% | +3 |  |  |  |  |
|  |  | <30% | +0 | Seizure duration ↘ | Yes / No | If “yes”: +1 |  |
|  |  |  |  | Seizure intensity ↘ | Yes / No | If “yes”: +1 |  |
|  |  |  |  | Post-ictal duration ↘ | Yes / No | If “yes”: +1 |  |
| **Total**  **(0-15)** |  | | | | | |  |

**Supplementary Table 2.** Individual CRRS for each participant. For some patients, magnet use could not be assessed because they were either seizure-free (3) or unable to perceive a seizure and unable to request assistance from a relative (4,6,8,13), indicated as NaN. To account of this, scores were calculated on a 10-point scale (only seizure frequency reduction) and subsequently rescaled to 15 using a rule-of-three conversion.

| **Pat ID** | **1** | **2** | **3** | **4** | **5** | **6** | **7** | **8** | **9** | **10** | **11** | **12** | **13** | **14** | **15** |
| --- | --- | --- | --- | --- | --- | --- | --- | --- | --- | --- | --- | --- | --- | --- | --- |
| **Binary Response** | R | R | R | R | R | R | R | NR | NR | NR | NR | NR | NR | NR | NR |
| **Acute- Magnet Response** | 5 | 5 | NaN | NaN | 4 | NaN | 5 | NaN | 5 | 5 | 5 | 4 | NaN | 4 | 5 |
| **Chronic** | 10 | 10 | 10 | 9 | 8 | 10 | 10 | 8 | 1 | 2 | 4 | 3 | 2 | 7 | 7 |
| **Total CRRS** | 15 | 15 | 15 | 13 | 12 | 13 | 15 | 12 | 6 | 7 | 9 | 7 | 2 | 11 | 12 |

**Supplementary Table 3.** Individual behavioral results. Mean and standard deviation of behavioral results for the whole patient cohort under each experimental condition. Reaction time is defined as the time taken by the patient to press the button, and the accuracy as the number of targets detected.

|  | | **VNS OFF** | | **VNS ON** | | **VNS ON LOW** | | **VNS ON HIGH** | |
| --- | --- | --- | --- | --- | --- | --- | --- | --- | --- |
| **Measure** | | Mean  Accuracy [%] | Response Time [s] | Mean  Accuracy [%] | Response Time [s] | Mean  Accuracy [%] | Response Time [s] | Mean  Accuracy [%] | Response Time [s] |
| **Pat ID** | |  |  |  |  |  |  |  |  |
| **1** | R | 97.62 | 0.397±  0.048 | 98.63 | 0.377 ± 0.122 | 96.00 | 0.339 ± 0.072 | 100 | 0.393 ± 0.135 |
| **2** | R | 100 | 0.362 ±  0.064 | 98.61 | 0.350 ± 0.065 | 96.15 | 0.353 ± 0.065 | 100 | 0.348 ± 0.074 |
| **3** | R | 95.24 | 0.623 ±  0.124 | 97.26 | 0.638 ± 0.280 | 96.97 | 0.656 ± 0.333 | 97.50 | 0.624 ± 0.253 |
| **4** | R | 97.62 | 0.488 ±  0.081 | 98.63 | 0.546 ± 0.193 | 96.77 | 0.578 ± 0.263 | 100 | 0.524 ± 0.151 |
| **5** | R | 97.62 | 0.342 ±  0.102 | 100 | 0.498 ± 0.233 | 100 | 0.500 ± 0.282 | 100 | 0.491 ± 0.212 |
| **6** | R | 97.62 | 0.427 ±  0.239 | 98.31 | 0.491 ± 0.331 | 96.00 | 0.495 ± 0.389 | 100 | 0.488 ± 0.305 |
| **7** | R | 100 | 0.369 ±  0.046 | 82.19 | 0.423 ± 0.097 | 86.49 | 0.416 ± 0.111 | 80.56 | 0.428 ± 0.099 |
| **8** | NR | 100 | 0.445 ±  0.114 | 98.63 | 0.419 ± 0.095 | 96.67 | 0.437 ± 0.135 | 100 | 0.406 ± 0.080 |
| **9** | NR | 100 | 0.316 ±  0.059 | 98.63 | 0.364 ± 0.055 | 96.00 | 0.365 ± 0.050 | 100 | 0.364 ± 0.065 |
| **10** | NR | 100 | 0.499 ±  0.060 | 98.63 | 0.487 ± 0.129 | 97.14 | 0.495 ± 0.174 | 100 | 0.480 ± 0.105 |
| **11** | NR | 97.62 | 0.374 ±  0.067 | 98.63 | 0.451 ± 0.111 | 96.67 | 0.451 ± 0.134 | 100 | 0.452 ± 0.106 |
| **12** | NR | 95.24 | 0.425 ±  0.083 | 98.63 | 0.456 ± 0.123 | 96.88 | 0.444 ± 0.122 | 100 | 0.465 ± 0.135 |
| **13** | NR | 97.62 | 0.371 ±  0.055 | 90.41 | 0.441 ± 0.114 | 94.44 | 0.432 ± 0.127 | 86.11 | 0.450 ± 0.120 |
| **14** | NR | 97.62 | 0.387 ±  0.076 | 98.63 | 0.363 ± 0.062 | 97.30 | 0.355 ± 0.050 | 100 | 0.372 ± 0.074 |
| **15** | NR | 100 | 0.425 ±  0.073 | 97.26 | 0.379 ± 0.108 | 94.59 | 0.397 ± 0.145 | 100 | 0.362 ± 0.082 |

**Supplementary Table 4.** Individual electrophysiological results of the P3b peak amplitude and peak latency for the whole patient cohort under each experimental condition. Peak amplitude is the amplitude corresponding to the amplitude of the highest peak in the 230-ms – 650-ms post-stimulus timeframe and is indicated for each condition on the left. Peak latency, on the right, is the corresponding time where this value is reached by the waveform.

|  | | **VNS OFF** | | **VNS ON** | | **VNS ON LOW** | | **VNS ON HIGH** | |
| --- | --- | --- | --- | --- | --- | --- | --- | --- | --- |
| **Measure** | | Peak Amplitude [µV] | Peak Latency  [s] | Peak Amplitude [µV] | Peak Latency  [s] | Peak Amplitude [µV] | Peak Latency  [s] | Peak Amplitude [µV] | Peak Latency  [s] |
| **PAT ID** | |  |  |  |  |  |  |  |  |
| **1** | R | 3.869 | 0.363 | 4.238 | 0.511 | 5.528 | 0.523 | 3.992 | 0.509 |
| **2** | R | 4.273 | 0.358 | 3.249 | 0.378 | 4.661 | 0.392 | 3.166 | 0.481 |
| **3** | R | 9.293 | 0.489 | 5.417 | 0.605 | 5.669 | 0.608 | 6.318 | 0.499 |
| **4** | R | 19.032 | 0.383 | 16.307 | 0.502 | 18.757 | 0.511 | 15.178 | 0.472 |
| **5** | R | 3.877 | 0.433 | 3.798 | 0.494 | 3.477 | 0.479 | 4.487 | 0.609 |
| **6** | R | 4.403 | 0.471 | 3.809 | 0.646 | 3.525 | 0.596 | 2.899 | 0.597 |
| **7** | R | 7.525 | 0.320 | 3.693 | 0.253 | 4.613 | 0.346 | 6.408 | 0.337 |
| **8** | NR | 7.209 | 0.532 | 8.811 | 0.355 | 7.931 | 0.354 | 9.614 | 0.355 |
| **9** | NR | 5.547 | 0.595 | 6.546 | 0.385 | 5.899 | 0.395 | 6.956 | 0.382 |
| **10** | NR | 8.363 | 0.386 | 8.895 | 0.398 | 8.531 | 0.403 | 9.222 | 0.396 |
| **11** | NR | 7.130 | 0.373 | 7.161 | 0.390 | 7.124 | 0.290 | 8.558 | 0.465 |
| **12** | NR | 10.312 | 0.364 | 11.451 | 0.393 | 13.534 | 0.400 | 10.051 | 0.431 |
| **13** | NR | 4.369 | 0.314 | 6.422 | 0.293 | 7.461 | 0.292 | 5.752 | 0.297 |
| **14** | NR | 10.777 | 0.404 | 17.408 | 0.371 | 19.173 | 0.365 | 17.361 | 0.407 |
| **15** | NR | 14.494 | 0.486 | 15.898 | 0.489 | 15.296 | 0.486 | 16.759 | 0.492 |

**Supplementary Table 5.** Parameters and results of the LMM. The dependent variable is the reaction time, defined as the average value of the time between the sound stimuli and the patient motor response recorded through a button. The model coefficient estimates with SE, t-statistics, confidence intervals, and p-values, p-values corrected using FDR, are reported for each condition by group of responses for the difference between each group within each condition, and for the interaction effect between groups and condition.

|  | | **Estimate** | **SE** | **t-Stat** | **Lower** | **Upper** | **p-val** | **P-FDR** |
| --- | --- | --- | --- | --- | --- | --- | --- | --- |
| **Condition** | | | | | | | | |
| [OFF] x ON | R | 0.045 | 0.015 | 3.093 | 0.016 | 0.074 | **0.003**** | **0.024*** |
|  | NR | 0.015 | 0.014 | 1.090 | −0.013 | 0.042 | 0.281 | 0.358 |
| [OFF] x LOW | R | 0.047 | 0.015 | 3.229 | 0.018 | 0.076 | **0.002**** | **0.016*** |
|  | NR | 0.0168 | 0.014 | 1.230 | −0.011 | 0.044 | 0.224 | 0.358 |
| [OFF] x HIGH | R | 0.041 | 0.015 | 2.814 | 0.012 | 0.070 | **0.007**** | **0.037*** |
|  | NR | 0.013 | 0.014 | 0.989 | −0.014 | 0.041 | 0.327 | 0.358 |
| [HIGH] x LOW | R | 0.006 | 0.015 | 0.415 | -0.023 | 0.035 | 0.680 | 0.680 |
|  | NR | 0.003 | 0.014 | 0.241 | -0.024 | 0.031 | 0.811 | 0.811 |
| **Response** | | | | | | | | |
| OFF | | -0.024 | 0.038 | -0.649 | -0.100 | 0.051 | 0.519 | 0.598 |
| ON | | −0.055 | 0.038 | −1.451 | −0.130 | 0.021 | 0.153 | 0.324 |
| LOW | | −0.055 | 0.038 | −1.453 | −0.130 | 0.021 | 0.152 | 0.324 |
| HIGH | | −0.052 | 0.038 | −1.379 | −0.128 | 0.024 | 0.174 | 0.348 |
| **Interaction** | | | | | | | | |
| [OFF] x ON | | -0.030 | 0.020 | -1.514 | -0.070 | 0.010 | 0.136 | 0.288 |
| [OFF] x LOW | | -0.030 | 0.020 | -1.518 | -0.070 | 0.010 | 0.135 | 0.288 |
| [OFF] x HIGH | | -0.028 | 0.020 | -1.379 | -0.068 | 0.013 | 0.174 | 0.348 |
| [HIGH] x LOW | | -0.003 | 0.020 | -0.139 | -0.043 | 0.037 | 0.890 | 0.890 |

**Supplementary Table 6.** Parameters and results of the LMM for the behavioral response for the conditions VNS OFF, VNS ON, VNS ON LOW, and VNS ON HIGH. The measure used in the model was the accuracy, defined as the percentage of targets detected by the patient and identified through a motor response. The model coefficient estimates with SE, t-statistics, confidence intervals, and p-values, p-values corrected using FDR, are reported for each condition by group of responses for the difference between each group within each condition, and for the interaction effect between groups and condition.

|  | | **Estimate** | **SE** | **t-Stat** | **Lower** | **Upper** | **p-val** | **P-FDR** |
| --- | --- | --- | --- | --- | --- | --- | --- | --- |
| **Condition** | | | | | | | | |
| [OFF] x ON | R | −1.727 | 1.525 | −1.133 | −4.788 | 1.333 | 0.263 | 0.934 |
|  | NR | −1.081 | 1.427 | −0.758 | −3.944 | 1.782 | 0.452 | 0.934 |
| [OFF] x LOW | R | −2.477 | 1.525 | −1.624 | −5.537 | 0.584 | 0.111 | 0.592 |
|  | NR | −2.302 | 1.427 | −1.613 | −5.164 | 0.561 | 0.113 | 0.592 |
| [OFF] x HIGH | R | −1.095 | 1.525 | −0.718 | −4.155 | 1.966 | 0.476 | 0.934 |
|  | NR | −0.249 | 1.427 | −0.174 | −3.111 | 2.614 | 0.862 | 0.934 |
| [HIGH] x LOW | R | -1.382 | 1.525 | -0.906 | -4.442 | 1.679 | 0.369 | 0.934 |
|  | NR | -2.053 | 1.427 | -1.439 | -4.916 | 0.810 | 0.156 | 0.747 |
| **Response** | | | | | | | | |
| OFF | | 0.553 | 2.037 | 0.271 | −3.534 | 4.639 | 0.787 | 0.934 |
| ON | | 1.199 | 2.037 | 0.589 | -2.888 | 5.286 | 0.559 | 0.934 |
| LOW | | 0.728 | 2.037 | 0.357 | -3.359 | 4.814 | 0.722 | 0.934 |
| HIGH | | 1.399 | 2.037 | 0.687 | -0.906 | 9.321 | 0.495 | 0.934 |
| **Interaction** | | | | | | | | |
| [OFF] x ON | | 0.646 | 2.088 | 0.310 | −3.544 | 4.837 | 0.758 | 0.934 |
| [OFF] x LOW | | 0.175 | 2.088 | 0.084 | −4.016 | 4.366 | 0.934 | 0.934 |
| [OFF] x HIGH | | 0.846 | 2.088 | 0.405 | −3.344 | 5.037 | 0.687 | 0.934 |
| [HIGH] x LOW | | -0.671 | 2.088 | -0.322 | -4.862 | 3.519 | 0.749 | 0.934 |

**Supplementary Table 7.** Parameters and results of the LMM for the electrophysiological response for the conditions VNS OFF, VNS ON, VNS ON LOW, and VNS ON HIGH. The measure used in the model was the P3b peak amplitude, defined as the amplitude of the highest peak in the 230-ms – 650-ms post-stimulus timeframe. The model coefficient estimates with SE, t-statistics, confidence intervals, and p-values, p-values corrected using FDR, are reported for each condition by group of responses for the difference between each group within each condition, and for the interaction effect between groups and condition.

|  | | **Estimate** | **SE** | **t-Stat** | **Lower** | **Upper** | **p-val** | **P-FDR** |
| --- | --- | --- | --- | --- | --- | --- | --- | --- |
| **Condition** | | | | | | | | |
| [OFF] x ON | R | -1.680 | 0.627 | -2.678 | -2.939 | -0.421 | **0.010**** | **0.023*** |
|  | NR | 1.799 | 0.587 | 3.065 | 0.621 | 2.977 | **0.004**** | **0.009**** |
| [OFF] x LOW | R | -0.864 | 0.627 | -1.376 | -2.122 | 0.396 | 0.175 | 0.233 |
|  | NR | 2.094 | 0.587 | 3.567 | 0.916 | 3.271 | **0.001***** | **0.004**** |
| [OFF] x HIGH | R | -1.404 | 0.627 | -2.237 | -2.663 | -0.145 | **0.030*** | 0.059 |
|  | NR | 2.009 | 0.587 | 3.423 | 0.831 | 3.187 | **0.001**** | **0.004**** |
| [HIGH] x LOW | R | 0.540 | 0.627 | 0.861 | -0.719 | 1.799 | 0.393 | 0.484 |
|  | NR | 0.085 | 0.587 | 0.144 | -1.093 | 1.262 | 0.886 | 0.886 |
| **Response** | | | | | | | | |
| OFF | | 1.058 | 2.203 | 0.480 | -3.363 | 5.478 | 0.633 | 0.675 |
| ON | | 4.537 | 2.203 | 2.059 | 0.116 | 8.957 | **0.045*** | 0.076 |
| LOW | | 4.015 | 2.203 | 1.822 | -0.406 | 8.435 | 0.074 | 0.108 |
| HIGH | | 4.470 | 2.203 | 2.029 | 0.050 | 8.891 | **0.048*** | 0.076 |
| **Interaction** | | | | | | | | |
| [OFF] x ON | | 3.479 | 0.859 | 4.050 | 1.755 | 5.203 | **>0.001***** | **0.002**** |
| [OFF] x LOW | | 2.957 | 0.859 | 3.442 | 1.233 | 4.681 | **0.001**** | **0.004**** |
| [OFF] x HIGH | | 3.413 | 0.859 | 3.972 | 1.689 | 5.137 | **>0.001***** | **0.002**** |
| [HIGH] x LOW | | -0.456 | 0.859 | -0.530 | -2.180 | 1.268 | 0.598 | 0.675 |

**Supplementary Table 8.** Parameters and results of the LMM for the electrophysiological response for the conditions VNS OFF, VNS ON, VNS ON LOW, and VNS ON HIGH. The measure used in the model was the P3b peak latency, defined as the time corresponding to the highest peak in the 230-ms – 650-ms post-stimulus timeframe. The model coefficient estimates with SE, t-statistics, confidence intervals, and p-values, p-values corrected using FDR, are reported for each condition by group of responses for the difference between each group within each condition, and for the interaction effect between groups and condition. The group of references is responders, and the condition of reference is indicated in brackets.

|  |  | **Estimate** | **SE** | **t-Stat** | **Lower** | **Upper** | **p-val** | **P-FDR** |
| --- | --- | --- | --- | --- | --- | --- | --- | --- |
| **Condition** | | | | | | | | |
| [OFF] x ON | R | 0.082 | 0.026 | 3.178 | 0.030 | 0.133 | **0.003**** | **0.007**** |
|  | NR | -0.048 | 0.024 | -1.976 | -0.096 | 0.001 | 0.054 | 0.078 |
| [OFF] x LOW | R | 0.091 | 0.026 | 3.550 | 0.040 | 0.143 | **0.001***** | **0.003**** |
|  | NR | -0.059 | 0.024 | -2.440 | -0.107 | -0.010 | **0.018*** | **0.032*** |
| [OFF] x HIGH | R | 0.098 | 0.026 | 3.821 | 0.047 | 0.150 | **>0.001***** | **0.003**** |
|  | NR | -0.029 | 0.024 | -1.193 | -0.077 | 0.020 | 0.238 | 0.293 |
| [HIGH] x LOW | R | -0.007 | 0.026 | -0.271 | -0.059 | 0.045 | 0.788 | 0.788 |
|  | NR | -0.030 | 0.024 | -1.246 | -0.078 | 0.018 | 0.218 | 0.291 |
| **Response** | | | | | | | | |
| OFF | | 0.029 | 0.041 | 0.713 | -0.053 | 0.112 | 0.479 | 0.547 |
| ON | | -0.100 | 0.041 | -2.438 | -0.182 | -0.018 | **0.018*** | **0.032*** |
| LOW | | -0.121 | 0.041 | -2.944 | -0.203 | -0.038 | **0.005**** | **0.011*** |
| HIGH | | -0.098 | 0.041 | -2.383 | -0.180 | -0.015 | **0.021*** | **0.033*** |
| **Interaction** | | | | | | | | |
| [OFF] x ON | | -0.129 | 0.035 | -3.670 | -0.200 | -0.059 | **0.001***** | **0.003**** |
| [OFF] x LOW | | -0.150 | 0.035 | -4.259 | -0.221 | -0.079 | **>0.001***** | **0.001**** |
| [OFF] x HIGH | | -0.127 | 0.035 | -3.605 | -0.198 | -0.056 | **0.001***** | **0.003**** |
| [HIGH] x LOW | | -0.023 | 0.035 | -0.654 | -0.094 | 0.048 | 0.516 | 0.551 |
