# Supplementary material for: Vagus Nerve Stimulation differentially modulates P3b in responders and non-responders: toward a biomarker of therapeutic efficacy
Source: Front Neurosci. 2026 Jun 17;20:1786262. doi: 10.3389/fnins.2026.1786262 (PMC13320354; doi:10.3389/fnins.2026.1786262)
Supplement: Supplementary file 2 [file Data_Sheet_2.DOCX]

Supplementary Material - 2

# Supplementary Data

**Detailed Results and Statistical Analysis of the Impact of Latency and Amplitude Estimation Measures**

This Supplementary Material presents and develops the methodology and biases induced by the choice of metric to assess both the amplitude and latency of the P3b.

Here, the individual values of another P3b amplitude metric, the P3b mean amplitude, defined as the average value of the target-non-target differential waveform in the 230ms – 650 ms post-stimulus timeframe are presented in Supplementary Table 1 along the P3b peak amplitude used in our work. The parameters and results of the LMM, including the model coefficient estimates with SE, t-statistics, confidence intervals, and p-values and p-values corrected using FDR are reported in Supplementary Table 3. Finally, bar plots of the mean value of P3b mean amplitude for each group under each condition are presented in Supplementary Figure 1.

Two additional measures were assed for the P3b latency: half peak latency and half-mean latency. Half-peak latency and half mean latency are the latencies corresponding to the moment where the target-non-target differential waveforms reach half of the values of the peak amplitude and mean latency, respectively. The individual results, along with the values for P3b peak latency, are reported in Supplementary Table 2. The parameters and results of the LMM, including the model coefficient estimates with SE, t-statistics, confidence intervals, and p-values and p-values corrected using FDR are reported in Supplementary Table 4 for half-peak latency and Supplementary Table 5 for half-mean latency. Finally, bar plots of the mean value of P3b mean amplitude for each group under each condition are presented in Supplementary Figure 2.

# Supplementary Figures and Tables

## Supplementary Figures


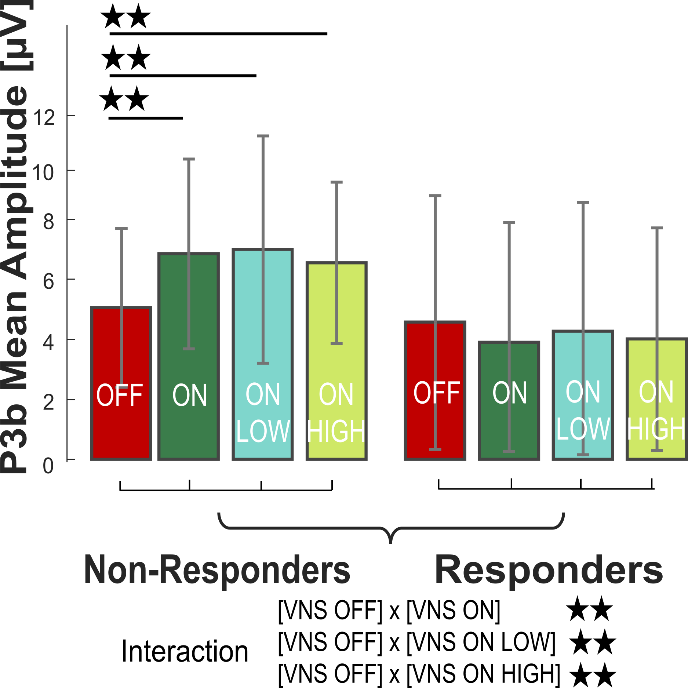


**Supplementary Figure 1.** Bar plots of mean amplitude of P3b, defined as the average value of the target-non-target differential ERP waveform in the 230 ms – 650-ms post-stimulus timeframe. The results are presented for each group, non-responders on the left and responders on the right, for VNS OFF in red, VNS ON in green, VNS ON LOW in turquoise and VNS ON HIGH in light green. The level of statistical significance is indicated with the following labels: (*) p ≤ 0.05, (**) p ≤ 0.01, (***) p ≤ 0.001.

**
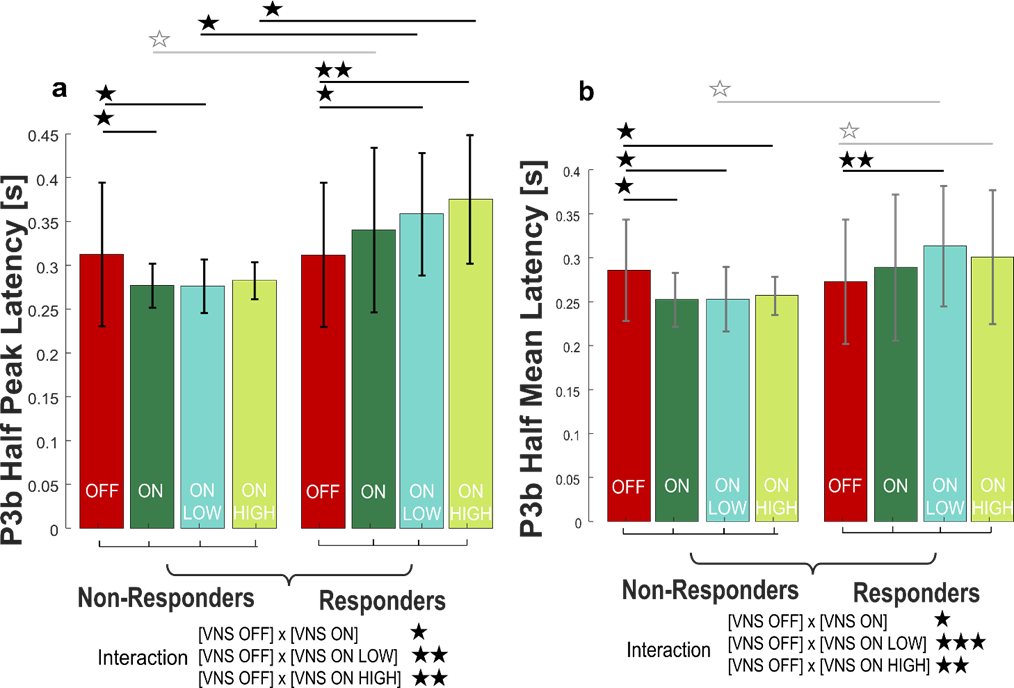
**

**Supplementary Figure 2.** Bar plots of half-peak latency of P3b and half-mean latency, defined as the latencies corresponding to the time where the target-non-target waveforms reach half of the values of the peak amplitude and mean amplitude, respectively. The results are presented for each group, non-responders on the left and responders on the right, for VNS OFF in red, VNS ON in green, VNS ON LOW in turquoise, and VNS ON HIGH in light green. The level of statistical significance is indicated with the following labels: : (*) p ≤ 0.05, (**) p ≤ 0.01, (***) p ≤ 0.001. The values significant before FDR correction are indicated by an empty star.

## Supplementary Tables

**Supplementary Table 1.** Electrophysiological results of the P3b amplitude for the whole patient cohort under each experimental condition. Peak amplitude is the amplitude corresponding to the amplitude of the highest peak in the 230 ms - 650 ms post-stimulus timeframe and is indicated for each condition on the left. Mean amplitude, on the right, corresponds to the average value of the waveform in the same timeframe.

|  | | **VNS OFF** | | **VNS ON** | | **VNS ON LOW** | | **VNS ON HIGH** | |
| --- | --- | --- | --- | --- | --- | --- | --- | --- | --- |
| **Measure**  **[µV]** | | Peak Amplitude | Mean Amplitude | Peak Amplitude | Mean Amplitude | Peak Amplitude | Mean Amplitude | Peak Amplitude | Mean Amplitude |
| **PAT ID** | |  |  |  |  |  |  |  |  |
| **1** | R | 3.869 | 1.887 | 4.238 | 2.388 | 5.528 | 3.393 | 3.992 | 2.123 |
| **2** | R | 4.273 | 1.362 | 3.249 | 2.076 | 4.661 | 3.112 | 3.166 | 1.415 |
| **3** | R | 9.293 | 5.889 | 5.417 | 3.130 | 5.669 | 2.617 | 6.318 | 3.645 |
| **4** | R | 19.032 | 13.379 | 16.307 | 12.911 | 18.757 | 13.927 | 15.178 | 11.949 |
| **5** | R | 3.877 | 2.392 | 3.798 | 2.742 | 3.477 | 1.986 | 4.487 | 3.173 |
| **6** | R | 4.403 | 2.246 | 3.809 | 1.978 | 3.525 | 1.869 | 2.899 | 1.268 |
| **7** | R | 7.525 | 4.844 | 3.693 | 2.072 | 4.613 | 2.944 | 6.408 | 4.540 |
| **8** | NR | 7.209 | 3.932 | 8.811 | 5.373 | 7.931 | 4.881 | 9.614 | 5.691 |
| **9** | NR | 5.547 | 2.169 | 6.546 | 4.434 | 5.899 | 3.320 | 6.956 | 4.957 |
| **10** | NR | 8.363 | 6.220 | 8.895 | 5.695 | 8.531 | 6.307 | 9.222 | 5.037 |
| **11** | NR | 7.130 | 4.644 | 7.161 | 5.361 | 7.124 | 4.674 | 8.558 | 5.764 |
| **12** | NR | 10.312 | 6.747 | 11.451 | 8.232 | 13.534 | 9.783 | 10.051 | 7.044 |
| **13** | NR | 4.369 | 1.070 | 6.422 | 2.969 | 7.461 | 3.076 | 5.7522 | 3.014 |
| **14** | NR | 10.777 | 6.419 | 17.408 | 11.728 | 19.173 | 13.276 | 17.361 | 9.849 |
| **15** | NR | 14.494 | 9.225 | 15.898 | 11.039 | 15.296 | 10.699 | 16.759 | 11.092 |

**Supplementary Table 2.** Electrophysiological results of the P3b latency for the whole patient cohort under each experimental condition. Peak latency is the latency corresponding to the latency of the highest peak in the 230 ms – 650 ms post-stimulus timeframe and is indicated for each condition on the left. Half-peak latency and half mean latency are the latencies corresponding to the moment where the target-non-target differential waveforms reach half of the values of the peak amplitude and mean latency, respectively.

|  | | **VNS OFF** | | | **VNS ON** | | | **VNS ON LOW** | | | **VNS ON HIGH** | | |
| --- | --- | --- | --- | --- | --- | --- | --- | --- | --- | --- | --- | --- | --- |
| **Measure [s]** | | Peak Latency | Half  Peak Latency | Half Mean Latency | Peak Latency | Half  Peak Latency | Half Mean Latency | Peak Latency | Half  Peak Latency | Half Mean Latency | Peak Latency | Half  Peak Latency | Half Mean Latency |
| **PAT ID** | |  |  |  |  |  |  |  |  |  |  |  |  |
| **1** | R | 0.363 | 0.311 | 0.299 | 0.511 | 0.399 | 0.355 | 0.523 | 0.374 | 0.359 | 0.509 | 0.407 | 0.392 |
| **2** | R | 0.358 | 0.319 | 0.287 | 0.378 | 0.336 | 0.323 | 0.392 | 0.346 | 0.329 | 0.481 | 0.437 | 0.321 |
| **3** | R | 0.489 | 0.354 | 0.296 | 0.605 | 0.398 | 0.376 | 0.608 | 0.438 | 0.393 | 0.499 | 0.396 | 0.374 |
| **4** | R | 0.383 | 0.302 | 0.285 | 0.502 | 0.287 | 0.267 | 0.511 | 0.298 | 0.272 | 0.472 | 0.276 | 0.263 |
| **5** | R | 0.433 | 0.365 | 0.274 | 0.494 | 0.347 | 0.229 | 0.479 | 0.392 | 0.295 | 0.609 | 0.352 | 0.243 |
| **6** | R | 0.471 | 0.391 | 0.346 | 0.646 | 0.451 | 0.334 | 0.596 | 0.419 | 0.357 | 0.597 | 0.471 | 0.334 |
| **7** | R | 0.320 | 0.139 | 0.121 | 0.253 | 0.165 | 0.139 | 0.346 | 0.241 | 0.189 | 0.337 | 0.287 | 0.179 |
| **8** | NR | 0.532 | 0.336 | 0.322 | 0.355 | 0.304 | 0.271 | 0.354 | 0.319 | 0.295 | 0.355 | 0.284 | 0.257 |
| **9** | NR | 0.595 | 0.488 | 0.387 | 0.385 | 0.309 | 0.296 | 0.395 | 0.316 | 0.300 | 0.382 | 0.308 | 0.297 |
| **10** | NR | 0.386 | 0.268 | 0.259 | 0.398 | 0.262 | 0.251 | 0.403 | 0.253 | 0.247 | 0.396 | 0.273 | 0.255 |
| **11** | NR | 0.373 | 0.250 | 0.242 | 0.390 | 0.259 | 0.246 | 0.290 | 0.251 | 0.244 | 0.465 | 0.285 | 0.247 |
| **12** | NR | 0.364 | 0.238 | 0.227 | 0.393 | 0.251 | 0.242 | 0.400 | 0.255 | 0.245 | 0.431 | 0.249 | 0.239 |
| **13** | NR | 0.314 | 0.258 | 0.228 | 0.293 | 0.257 | 0.246 | 0.292 | 0.256 | 0.244 | 0.297 | 0.258 | 0.247 |
| **14** | NR | 0.404 | 0.311 | 0.289 | 0.371 | 0.268 | 0.191 | 0.365 | 0.257 | 0.182 | 0.407 | 0.294 | 0.231 |
| **15** | NR | 0.486 | 0.350 | 0.333 | 0.489 | 0.306 | 0.275 | 0.486 | 0.304 | 0.267 | 0.492 | 0.308 | 0.281 |

**Supplementary Table 3.** Groups' electrophysiological responses (mean amplitude, half-peak latency and half-mean latency) and LMM analysis results. Mean values and standard deviation of behavioral and electrophysiological results for the VNS OFF, VNS ON, VNS ON LOW, and VNS ON HIGH conditions. Results from the Type III ANOVA with Satterthwaite’s approximation applied to the linear mixed model are reported for the main effects of Condition, Response, and their interaction. The level of statistical significance was set at p < 0.05 and indicated with *, p < 0.01 with ** and p < 0.001 with ***.

|  | **Electrophysiological Results** | | | | | **LMM Statistics** | | |
| --- | --- | --- | --- | --- | --- | --- | --- | --- |
|  |  | **VNS OFF** | **VNS ON** | **VNS ON LOW** | **VNS ON HIGH** | **Condition** | **Response** | **Interaction** |
| **Mean Amplitude [µV]** | **R** | **4.571 ± 4.224** | **3.900 ± 3.995** | **4.264 ± 4.298** | **4.016 ± 3.696** | **p = 0.149** | **p = 0.211** | **p = 0.004**** |
|  | **NR** | **5.053 ± 2.649** | **6.854 ± 3.160** | **7.002 ± 3.782** | **6.556 ± 2.684** |  |  |  |
| **Half-Peak Latency [s]** | **R** | **0.312 ± 0.083** | **0.340 ± 0.094** | **0.358 ± 0.069** | **0.375 ± 0.074** | **p = 0.318** | **p = 0.049*** | **p < 0.001***** |
|  | **NR** | **0.312 ± 0.082** | **0.277 ± 0.025** | **0.276 ± 0.031** | **0.282 ± 0.022** |  |  |  |
| **Half-Mean Latency [s]** | **R** | **0.273 ± 0.071** | **0.289 ± 0.083** | **0.313 ± 0.068** | **0.301 ± 0.076** | **p = 0.505** | **p = 0.241** | **p < 0.001***** |
|  | **NR** | **0.286 ± 0.058** | **0.252 ± 0.031** | **0.253 ± 0.037** | **0.257 ± 0.022** |  |  |  |

**Supplementary Table 4.** Results of the LMM for the electrophysiological response for the conditions VNS OFF, VNS ON, VNS ON LOW, and VNS ON HIGH. The measure used in the model was P3b mean amplitude, defined as the average value of the target-non-target differential waveform in the 230-ms – 650-ms post-stimulus timeframe. The model coefficient estimates with SE, t-statistics, confidence intervals, and p-values, p-values corrected using FDR, are reported for each condition by group of responses for the difference between each group within each condition, and for the interaction effect between groups and condition. The group of references is responders, and the condition of reference is indicated in brackets.

|  | | **Estimate** | **SE** | **t-Stat** | **Lower** | **Upper** | **p-val** | **p-FDR** |
| --- | --- | --- | --- | --- | --- | --- | --- | --- |
| **Condition** | | | | | | | | |
| [OFF] x ON | R | -0.672 | 0.519 | -1.295 | -1.713 | 0.369 | 0**.**201 | 0.321 |
|  | NR | 1.801 | 0.485 | 3.711 | 0.827 | 2.774 | **0.001***** | **0.004**** |
| [OFF] x LOW | R | -0.307 | 0.519 | -0.592 | -1.348 | 0.734 | 0.556 | 0.685 |
|  | NR | 1.949 | 0.485 | 4.016 | 0.975 | 2.922 | **>0.001***** | **0.002**** |
| [OFF] x HIGH | R | -0.555 | 0.519 | -1.070 | -1.596 | 0.486 | 0.289 | 0.421 |
|  | NR | 1.503 | 0.485 | 3.097 | 0.529 | 2.476 | **0.003**** | **0.010**** |
| [HIGH] x LOW | R | 0.248 | 0.519 | 0.478 | -0.793 | 1.289 | 0.635 | 0.725 |
|  | NR | 0.446 | 0.485 | 0.919 | -0.528 | 1.420 | 0.362 | 0.483 |
| **Response** | | | | | | | | |
| OFF | | 0.482 | 1.723 | 0.280 | -2.976 | 3.940 | 0.781 | 0.781 |
| ON | | 2.954 | 1.723 | 1.714 | -0.504 | 6.413 | 0.093 | 0.211 |
| LOW | | 2.738 | 1.723 | 1.589 | -0.720 | 6.196 | 0.118 | 0.236 |
| HIGH | | 2.540 | 1.723 | 1.474 | -0.918 | 5.998 | 0.147 | 0.260 |
| **Interaction** | | | | | | | | |
| [OFF] x ON | | 2.472 | 0.710 | 3.481 | 1.047 | 3.898 | **0.001***** | **0.005**** |
| [OFF] x LOW | | 2.256 | 0.710 | 3.176 | 0.831 | 3.681 | **0.003**** | **0.010**** |
| [OFF] x HIGH | | 2.058 | 0.710 | 2.897 | 0.633 | 3.483 | **0.006**** | **0.015**** |
| [HIGH] x LOW | | 0.198 | 0.710 | 0.279 | -1.227 | 1.623 | 0.781 | 0.781 |

**Supplementary Table 5.** Results of the LMM for the electrophysiological response for the conditions VNS OFF, VNS ON, VNS ON LOW, and VNS ON HIGH. The measure used in the model was P3b half-peak latency, defined as the time at which the target-non-target differential waveforms reach half of the values of peak amplitude detected in the 230-ms – 650-ms post-stimulus timeframe. The model coefficient estimates with SE, t-statistics, confidence intervals, and p-values, p-values corrected using FDR, are reported for each condition by group of responses for the difference between each group within each condition, and for the interaction effect between groups and condition. The group of references is responders, and the condition of reference is indicated in brackets.

|  | | **Estimate** | **SE** | **t-Stat** | **Lower** | **Upper** | **p-val** | **p-FDR** |
| --- | --- | --- | --- | --- | --- | --- | --- | --- |
| **Condition** | | | | | | | | |
| [OFF] x ON | R | 0.029 | 0.017 | 1.717 | -0.005 | 0.062 | 0.092 | 0.123 |
|  | NR | -0.036 | 0.016 | -2.278 | -0.067 | -0.004 | **0.027*** | **0.048*** |
| [OFF] x LOW | R | 0.047 | 0.017 | 2.802 | 0.013 | 0.080 | **0.007**** | **0.019*** |
|  | NR | -0.036 | 0.016 | -2.312 | -0.067 | -0.005 | **0.025*** | **0.048*** |
| [OFF] x HIGH | R | 0.063 | 0.017 | 3.810 | 0.030 | 0.097 | **>0.001***** | **0.003**** |
|  | NR | -0.030 | 0.016 | -1.914 | -0.061 | 0.001 | 0.061 | 0.089 |
| [HIGH] x LOW | R | -0.017 | 0.017 | -1.008 | -0.050 | 0.017 | 0.318 | 0.392 |
|  | NR | -0.006 | 0.016 | -0.398 | -0.037 | 0.025 | 0.693 | 0.739 |
| **Response** | | | | | | | | |
| OFF | | 0.001 | 0.031 | 0.017 | -0.062 | 0.063 | 0.986 | 0.986 |
| ON | | -0.064 | 0.031 | -2.044 | -0.126 | -0.001 | **0.046*** | 0.074 |
| LOW | | -0.082 | 0.031 | -2.643 | -0.145 | -0.020 | **0.011*** | **0.025*** |
| HIGH | | -0.093 | 0.031 | -2.983 | -0.155 | -0.030 | **0.004**** | **0.017*** |
| **Interaction** | | | | | | | | |
| [OFF] x ON | | -0.064 | 0.023 | -2.810 | -0.110 | -0.018 | **0.007**** | **0.019*** |
| [OFF] x LOW | | -0.083 | 0.023 | -3.626 | -0.128 | -0.037 | **0.001***** | **0.004**** |
| [OFF] x HIGH | | -0.093 | 0.023 | -4.090 | -0.139 | -0.048 | **>0.001***** | **0.003**** |
| [HIGH] x LOW | | 0.011 | 0.023 | 0.464 | -0.035 | 0.056 | 0.644 | 0.736 |

**Supplementary Table 6.** Results of the LMM for the electrophysiological response for the conditions VNS OFF, VNS ON, VNS ON LOW, and VNS ON HIGH. The measure used in the model was P3b half-mean latency, defined as the time at which the target-non-target differential waveforms reach half of the values of mean amplitude computed in the 230-ms – 650-ms post-stimulus timeframe. The model coefficient estimates with SE, t-statistics, confidence intervals, and p-values, p-values corrected using FDR, are reported for each condition by group of responses for the difference between each group within each condition, and for the interaction effect between groups and condition. The group of references is responders, and the condition of reference is indicated in brackets.

|  | | **Estimate** | **SE** | **t-Stat** | **Lower** | **Upper** | **p-val** | **p-FDR** |
| --- | --- | --- | --- | --- | --- | --- | --- | --- |
| **Condition** | | | | | | | | |
| [OFF] x ON | R | 0.016 | 0.012 | 1.324 | -0.008 | 0.041 | 0.191 | 0.265 |
|  | NR | -0.034 | 0.012 | -2.904 | -0.057 | -0.010 | **0.005**** | **0.017*** |
| [OFF] x LOW | R | 0.041 | 0.012 | 3.312 | 0.016 | 0.066 | **0.002**** | **0.009**** |
|  | NR | -0.033 | 0.012 | -2.832 | -0.056 | -0.010 | **0.007**** | **0.018*** |
| [OFF] x HIGH | R | 0.028 | 0.012 | 2.293 | 0.004 | 0.053 | **0.026*** | 0.052 |
|  | NR | -0.029 | 0.012 | -2.506 | -0.052 | -0.006 | **0.015*** | **0.035*** |
| [HIGH] x LOW | R | 0.013 | 0.012 | 1.018 | -0.012 | 0.037 | 0.313 | 0.386 |
|  | NR | -0.004 | 0.012 | -0.326 | -0.027 | 0.019 | 0.746 | 0.746 |
| **Response** | | | | | | | | |
| OFF | | 0.013 | 0.028 | 0.472 | -0.043 | 0.070 | 0.639 | 0.681 |
| ON | | -0.037 | 0.028 | -1.302 | -0.093 | 0.020 | 0.199 | 0.265 |
| LOW | | -0.060 | 0.028 | -2.145 | -0.117 | -0.004 | **0.037*** | 0.065 |
| HIGH | | -0.044 | 0.028 | -1.564 | -0.101 | 0.013 | 0.124 | 0.198 |
| **Interaction** | | | | | | | | |
| [OFF] x ON | | 0.050 | 0.017 | 2.951 | 0.016 | 0.084 | **0.005**** | **0.017*** |
| [OFF] x LOW | | 0.074 | 0.017 | 4.353 | 0.040 | 0.108 | **>0.001***** | **>0.001***** |
| [OFF] x HIGH | | 0.057 | 0.017 | 3.387 | 0.023 | 0.091 | **0.001***** | **0.009**** |
| [HIGH] x LOW | | -0.016 | 0.017 | -0.967 | -0.050 | 0.018 | 0.338 | 0.387 |
